# Deciphering Molecular Pathways of *Bletilla striata* Seeds Symbiotic Germination with *Tulasnella* sp. bj1

**DOI:** 10.3390/microorganisms14010174

**Published:** 2026-01-13

**Authors:** Yueyu Ye, Yucong Zhao, Ning Wang, Ruonan Tang, Zixin Huang, Shiqing Li, Meiya Li, Chunchun Zhang, Fusheng Jiang

**Affiliations:** 1 College of Pharmaceutical Sciences, Zhejiang Chinese Medical University, Hangzhou 310053, China; yyy13626675378@163.com (Y.Y.); 13819106998@163.com (Y.Z.); 15064488821@163.com (N.W.); hzxin7@163.com (Z.H.); 888lishiqing@163.com (S.L.); lmeiya@126.com (M.L.); 2 School of Pharmacy, Nanjing University of Chinese Medicine, Nanjing 210023, China; 3 College of Life Sciences, Zhejiang Chinese Medical University, Hangzhou 310053, China; trn000920@163.com; 4 Academy of Chinese Medical Sciences, Zhejiang Chinese Medical University, Hangzhou 310053, China

**Keywords:** *Bletilla striata*, symbiosis germination, flavonoids, jasmonic acid, indoleacetic acid

## Abstract

Orchid seed germination requires symbiotic association with mycorrhizal fungi that provide essential nutrients for germination and subsequent growth. Extensive research has elucidated the pivotal role of the mycorrhizal fungus *Tulasnella* sp. in the modulation of seed germination and growth processes in *Bletilla striata* (Thunb.) Reiehb.f. However, the molecular mechanisms underlying this symbiosis remain poorly characterized. Our integrated transcriptomic-metabolomic analysis of symbiotic germination revealed that co-cultivation of *Tulasnella* sp. bj1 with *B. striata* seeds significantly downregulates the expression of plant-derived flavonoid biosynthetic genes, with flavonoid degradation potentially alleviating germination and growth inhibition. The bj1 strain modulates indoleacetic acid (IAA) biosynthesis in *B. striata* by upregulating the expression of plant-derived tryptophan decarboxylase (TDC) in the tryptophan pathway and hydrolytic enzymes (NtAMI) in the indoleacetamide pathway, with elevated IAA potentially contributing to seed germination and growth. Moreover, bj1 suppresses the jasmonic acid (JA) biosynthetic pathway of *B. striata* by downregulating key plant-derived biosynthetic genes, concurrently promoting the accumulation of 12-hydroxyjasmonic acid—a metabolite associated with plant immune regulation that may favor colonization and symbiotic establishment with *B. striata* seeds. Additionally, bj1 induces the expression of polysaccharide-degrading enzymes, potentially improving carbon source utilization to support protocorm development. In conclusion, bj1 modulates the immune response of *B. striata* seeds, facilitating the establishment of a symbiotic relationship. Subsequently, the germination and growth of *B. striata* seeds are enhanced through reduced flavonoid accumulation, increased IAA synthesis, and improved carbon source utilization. Consequently, this investigation provides a crucial foundation for elucidating mechanisms governing symbiotic germination in *B. striata*.

## 1. Introduction

Seed germination marks the initiation of the plant life cycle, encompassing an ordered sequence of physiological processes and morphogenesis commencing with seed imbibition. The endosperm of most plant seeds serves as a significant reservoir of carbohydrates, proteins, lipids and minerals, providing essential nutrients for germination and subsequent growth and development [1,2,3]. In contrast, the Orchidaceae, the second largest family of angiosperms, produce minute seeds that lack both cotyledons and endosperm. These seeds comprise only an undifferentiated, primitive embryo with inadequate nutritional reserves [3]. Consequently, their autotrophic capacity for germination and complete ontogenetic development is severely limited [4]. Interestingly, the colonization of orchid seeds by certain fungi can facilitate their germination and growth, and eventually form plants [5]. The symbiotic association established between these fungi and the orchid root system is referred to as orchid mycorrhiza (OM) [6]. Orchid protocorms can acquire nutrients from both living and degraded hyphae of OM [4,7]. This distinctive method of nutrient acquisition also enables orchids to occupy a specialized niche in the ecosystem.

*Bletilla striata* (Thunb.) Reichb.f. (*B. striata*), an herbaceous orchid species of significant medicinal value, faces constraints in its development and utilization due to limited natural propagation capacity and resource scarcity [8]. Recent advances in symbiotic cultivation research have demonstrated that mycorrhizal fungi play a crucial role in enhancing seed germination and early development of this orchid [9]. For instance, Jiang et al. reported that the fungal strain *Fusarium oxysporum* KB-3 facilitates *B. striata* seed germination [8]. Further investigations by Xi et al. isolated strain R6 from wild *B. striata* seedlings [10]; this strain exhibits 96% ITS sequence similarity with *Serendipita indica* and demonstrates significant germination enhancement capabilities. Consequently, the symbiotic germination using mycorrhizal fungi with *B. striata* seeds presents a promising approach for increasing yield and supporting the sustainable development of the *B. striata* industry.

Flavonoids, compounds structurally derived from the 2-phenylchromanone backbone, are ubiquitous in plants, occurring in flowers, fruits, roots, and seeds [11]. Extensive studies have highlighted their critical physiological roles, including promoting growth and development, enhancing resistance to pathogens, deterring herbivores, and providing UV protection [12,13]. Flavonoids are also notably abundant in plant seeds; for instance, in the model organism *Arabidopsis thaliana*, flavonoid components accumulate significantly in the seed coat, particularly in the endothelium [14]. Flavonoids localized in the seed coat not only contribute to seed pigmentation but also play an essential role in maintaining seed viability and ensuring high germination capacity during desiccation and storage [15]. Conversely, abnormal accumulation of flavonoids in the seed coat [16] or embryo [17] can adversely affect seed development and vitality. Furthermore, plants can secrete flavonoids to directly influence neighboring plant seeds or suppress the germination of competing seeds by modulating the rhizosphere microbial community [18]. Nevertheless, relatively limited attention has been devoted to investigating the cumulative alterations in flavonoids and their underlying regulatory mechanisms during the seed germination and growth stages of orchids, particularly those modulated by mycorrhizal fungi. Therefore, elucidating the changes in flavonoids in orchid seeds offers novel perspectives for investigating the symbiotic germination mechanisms between mycorrhizal fungi and orchid seeds.

Orchid seeds rely on specific fungi for development from germination through protocorm formation to seedling establishment [19]. To explore mycelial colonization, bidirectional nutrient exchange, and molecular interactions between fungi and orchids, extensive research has been conducted, contributing to our understanding of symbiotic germination [3]. Studies demonstrate that OM fungi typically colonize the protocorm base. The mycelium penetrates protocorm tissues, forming intracellular pelotons that facilitate nutrient exchange [20]. OM fungi supply carbohydrates, such as alginate and glucose, and nitrogen to orchids, while orchid plants provide photosynthetic products to the fungal symbionts, establishing mutualism [3]. In addition, pelotons are not permanent structures; they are subsequently digested and absorbed by protocorm cells, providing nutrients for growth [21]. Beyond facilitating material exchange, OM fungi also promote protocorm development by secreting plant hormones [6]. However, it remains unclear how OM fungi recognize specific orchid seeds and whether seed-secreted chemical signals induce mycelial colonization. Additionally, changes in host immunity during OM fungal infestation of seeds are rarely documented in existing literature.

The research team had previously verified through field experiments that the mycorrhizal fungus *Tulasnella* sp. bj1 strain can promote the growth of *B. striata* plants, enhance the accumulation of bioactive compounds such as polysaccharides and resveratrol in tubers, and upregulate the expression of relevant genes, thereby improving the quality of the traditional Chinese medicine *B. striata* [22]. However, there has been a lack of systematic research on how bj1 effectively promotes the germination and growth of *B. striata* seeds. In the current study, metabolomics and transcriptomics were utilized to carry out a systematic analysis of the metabolites and gene expression of *B. striata* seeds at different times during symbiotic germination. This study focuses on investigating the mechanisms underlying immune responses, flavonoid biosynthesis, and plant hormone synthesis during the germination of *B. striata* seeds. The results suggest that the bj1 may contribute to the degradation of flavonoids and reduce their biosynthesis, based on the observed decline in flavonoid contents over time and lower levels in the bj1-treated group—changes that could be conducive to the germination and growth of *B. striata* seeds. Consistent with prior studies, symbiotic germination of *B. striata* seeds with bj1 elevated IAA synthesis-related gene expression and IAA accumulation, while also inducing polysaccharide-degrading genes to support carbon utilization and growth of *B. striata* protocorms. Additionally, we also noticed that in the early stage of bj1 colonization, the contents of some components with antibacterial activity decreased significantly, which might be conducive to the establishment of the symbiotic relationship between bj1 and *B. striata* seeds. These results provided new insights into the mechanism of establishing the symbiotic relationship between the mycorrhizal fungus bj1 and *B. striata* seeds and facilitating their growth and germination.

## 2. Materials and Methods

### 2.1. Plant and Mycorrhizal Fungus Materials

The OM fungus strain bj1 (*Tulasnella* sp., NCBI accession: OR245559), previously isolated from the rhizomes of *B. striata* and identified in a prior study [22], was maintained on solid potato dextrose agar (PDA) medium (containing 200 g/L potato, 20 g/L glucose, and 15 g/L agar, pH 5.2). The strain is preserved at the China General Microbiological Culture Collection Center under accession number 40,773. To isolate seed germination responses from PDA-derived nutrients, we developed a novel assay in this study: PDA plates were overlaid with a thin nutrient-free agar layer, with only the central fungal inoculation point exposed. This approach eliminates confounding effects of PDA nutrients on seed germination. The plates were then cultivated at 25 °C for 6 days until complete mycelial coverage was achieved. *B. striata* seeds were collected from Yujiashan Village, Linpuzhen, Xiaoshan District, Hangzhou, China (30.03507° N, 120.22744° E). Fruit pods were collected in October 2025, coinciding with the stage of full seed maturity. Only pods exhibiting intact surfaces and no visible signs of damage were selected [23]. Immediate surface disinfection was conducted following collection. Five pods were harvested from each of 10 individual plants, resulting in a total of 50 pods. Seeds from multiple pods were pooled for sampling and subsequent inoculation. Following disinfection, all samples were stored under controlled environmental conditions to maintain viability. The seed samples are stored in the germplasm bank of the Institute of Traditional Chinese Medicine Resources at Zhejiang Chinese Medical University. Seed capsules were disinfected with 75% ethanol for 5 min followed by conventional bleach (4% sodium hypochlorite composition) for 7 min, then rinsed six times with sterile water. Post-disinfection, seeds were aseptically transferred onto the dual-layer system containing pre-colonized bj1 mycelia (bj1 group), while non-inoculated dual-layer PDA plates served as controls. Sow 100–150 seeds in each Petri dish, with 5 biological replicates per experimental group. All samples were placed in an incubator and cultivated at 25 °C under a 16 h light/8 h dark photoperiod. According to the growth stage evaluation criteria established by Stewart et al. [24] for *B. striata*, samples were collected at 7 days (Stage 2), 14 days (Stage 3), 21 days (Stage 4), and 28 days (Stage 5) post-inoculation. Collected samples were immediately stored at −80 °C for subsequent analyses.

### 2.2. Fungal Colonization

The success of fungal colonization was assessed using the staining method described by Jiang et al. [8]. Thirty *B. striata* protocorms were randomly selected from each group for examination. The presence of pelotons confirmed that the mycorrhizal fungus had successfully colonized, and the colonization rate was subsequently calculated.

### 2.3. Metabolomics Study

#### 2.3.1. Samples

The protocorm samples of control group and bj1 group were collected and homogenized with a tissue homogenizer. The samples were dried in a freeze-vacuum desiccator (VirTis, AD2.0 EL, SP Scientific, Warminster, PA, USA), after which 10 mg of each sample was accurately weighed and added to 250 μL of 50% ethanol. Subsequently, the samples were incubated at room temperature for 30 min, followed by sonication for 15 min. After centrifugation (12,000 rpm, 4 °C, 5 min), 200 μL of the supernatant was carefully aspirated and transferred into a new vial for UPLC-MS/MS analysis. All samples were combined with the same amount of supernatant as a quality control (QC) sample, and 50% ethanol was used as the blank sample. Subsequently, the positive ion mode (POS) and negative ion mode (NEG) were recorded.

#### 2.3.2. LC-MS Conditions

Samples were injected into an ACQUITY ultraperformance liquid chromatography instrument (Waters Corporation, Milford, MA, USA). Samples were separated by a Waters ACQUITY UPLC Cortecs T3 column (2.1 mm × a100 mm, 1.7 µm, Waters, UK) at a column temperature of 30 °C and a flow rate of 0.35 mL/min, with acetonitrile (A) and 0.1% formic acid (B) serving as solvents. The gradient elution program was as follows: 0–3 min, 5% A; 3–6 min, 5–10% A; 6–16 min, 10–20% A; 16–20 min, 20–25% A; 20–28.5 min, 25–42% A; 28.5–44.5 min, 42–85% A; 44.5–46.5 min, 85–100% A; 46.5–49 min, 100% A; 49–51 min, 100–5% A; and 51–54 min, 5% A. A SYNAPT G2-Si QTOF Mass Spectrometer (Waters Corporation, Manchester, UK) was used to obtain the data. Both positive and negative ion modes were used to operate the source, and the capillary voltage was set at 2.51 kV. The entire scan mass was carried out between *m*/*z* 50 and 1500.

#### 2.3.3. Metabolite Identification and Analysis

The raw data were preprocessed by Progenesis QI v2.3 (Nonlinear, Dynamics, Newcastle, UK), with the precursor and product tolerance set at 5 and 10 ppm, respectively, and the production threshold set at 5%. A qualitative identification of the chemical was carried out based on the compound’s reported secondary fragments, isotope distribution, MONA-export-LC-MS database, and ChemSpider database. More than 50% of the extracted data were further processed, and compounds with scores < 36 (full score of 60) were regarded as inaccurate qualitative results and deleted. The Masslyn software (MassLynx Version 4.1) was utilized to compare the first-order accurate mass-to-charge ratio and second-order fragment information for compound determination. In addition, compounds with high scores but peak areas below 500 were removed to mitigate low-abundance measurement artifacts. The LC-MS data matrix was imported into SIMCA14.1 software (Version 14.1) for principal component analysis (PCA), ensuring sample stability throughout the analysis process. Orthogonal partial least squares discriminant analysis (OPLS-DA) was conducted to discern overall differences in metabolic spectra between the groups. The OPLS-DA model was utilized to obtain the variable importance in projection (VIP) values. Among them, metabolites with fold change (FC) > 2, t-test *p*-value < 0.05, VIP > 1, false discovery rate (FDR) ≤ 0.05 were identified as differentially abundant metabolites (DAMs). These DAMs were subsequently mapped to the KEGG metabolic pathways (https://www.kegg.jp/, accessed on 1 December 2024) for pathway enrichment analysis.

### 2.4. Transcriptomic Analysis

Total RNA was isolated from both groups using a plant-specific RNA extraction kit, followed by quality assessment with an Agilent 2100 Bioanalyzer (Agilent Technologies, Santa Clara, CA, USA) to verify RNA integrity and quantity. mRNA was enriched from total RNA using oligo(dT) magnetic beads (Thermo Fisher Scientific, Waltham, MA, USA), which specifically bind to the poly(A) tails of eukaryotic mRNA, thereby enabling the separation of mRNA from rRNA, tRNA, and other non-coding RNA species. RNA libraries were prepared using a Qubit 2.0 Fluorometer (Thermo Fisher Scientific) for precise quantification and normalized to 1.5 ng/μL. Library fragment size distribution was validated via Agilent 2100 analysis prior to paired-end sequencing (2 × 150 bp) on the Illumina NovaSeq™ 6000 platform (Renke Biotechnology Co., Ltd., Shanghai, China). Clean reads were spliced using Trinity software (Version 2.14.0), where the parameter min_kmer_cov was set to 2, and the spliced results were stored in FASTA format. The longest transcript of each gene was selected as Unigene by the length statistics of the spliced transcript sequence, and the unigene sequence was functionally annotated. The unigene sequences were annotated for gene functions in seven major databases, including the evolutionary genealogy of genes: Non-supervised Orthologous Groups (eggNOG), NCBI Non-Redundant Protein Database (NCBI_NR), Pfam database, Swiss-Prot Protein Sequence Database (Swiss-Prot), Kyoto Encyclopedia of Genes and Genomes (KEGG), and Gene Ontology database (GO). The transcripts per kilobase of exon model per million mapped reads (TPM) values of the samples were calculated using RSEM software (Version 1.2.31). Differentially expressed genes (DEGs) between groups were identified by comparing the expression differences in component RNA using DESeq2 software (Version 1.30) with a false discovery rate (FDR) < 0.05, Flodchange > 2, and a *p*-adjusted < 0.05 [22]. The groups of differential genes included *B. striata* seed 7 d bj1 group and control group (bj1 w1 vs. control w1, with W = Week), 14 d bj1 group and control group (bj1 w2 vs. control w2). Enrichment analyses of DEGs were subsequently performed using GO term (http://geneontology.org/ accessed on 5 December 2024) and KEGG databases to identify significantly regulated biological processes, metabolic pathways, and signaling cascades. To elucidate the relationship between metabolites and genes in the aforementioned pathways, we integrated the differential metabolites and genes associated with flavonoids and plant hormones to construct a Pearson correlation analysis network (|r| > 0.8, *p* < 0.05).

### 2.5. Quantitative Real-Time PCR (qPCR) Analysis

The expression of genes related to flavonoid, starch and sucrose metabolic, and phytohormone biosynthesis pathways in *B. striata* protocorms was verified by qPCR using GAPDH as an internal reference gene [25]. The primer sequences of each target gene are presented in Supplementary Table S1. Total RNA from *B. striata* protocorms were extracted using a TaKaRa MiniBEST Plant RNA Extraction Kit (TaKaRa, Dalian, China). The selected kit is Protocol-I, which employs a streamlined tissue extraction procedure. The starting material is 100 mg of plant tissue. The workflow comprises tissue lysis, column-based binding and purification, on-column DNase digestion. cDNA was synthesized from 2 μg of isolated mRNA using 4 μL of CoWin’s HiFi Script gDNA Remote RT MasterMix (CoWin, Nanjing, China) in a 20 μL reaction volume: the protocol followed 42 °C for 30 min (reverse transcription) and 85 °C for 5 min (enzyme inactivation). qPCR was conducted on a Light Cycler 96 instrument (Roche, Mannheim, Germany) in a 20 μL system: 10 μL of 2× SYBR Green qPCR MasterMix, 0.4 μL of each 10 μM forward/reverse primer, 2 μL of cDNA template, and 7.2 μL of RNase-free water. Cycling conditions: pre-denaturation at 95 °C for 3 min, 40 cycles of 95 °C (10 s, denaturation) and 60 °C (30 s, annealing/extension). Each sample included 3 technical replicates, with ≥3 biological replicates per group. The relative gene expression level was calculated using the 2^−(ΔΔCT)^ method [25]. The method employs a subtraction strategy based on two sequential cycle threshold (CT) differentials. ΔCT was calculated for each sample as the CT difference between the target gene and internal reference, eliminating sample-specific inconsistencies. ΔΔCT was derived by subtracting the mean ΔCT of the control group from each experimental group sample’s ΔCT, normalizing experimental expression to the control baseline.

### 2.6. Statistical Analysis

GraphPad Prism 8.0 (GraphPad Software, La Jolla, CA, USA) was used for data processing, statistical analysis, and plotting. All data are expressed as mean ± standard deviation. A two-way analysis of variance (ANOVA) was conducted to compare samples among different treatment groups and time points (measured in weeks). An independent-samples *t*-test was adopted to analyze the differences between the two groups. *p* ˂ 0.05 was considered statistically significant. Significant differences between groups are indicated by different letters.

## 3. Results

### 3.1. Effective Colonization by bj1 and Metabolomics Analysis of B. striata Protocorms

Protocorms of *B. striata* co-cultured with bj1 for 14 days exhibited significantly larger sizes compared to the non-symbiotic group (Supplementary Figure S1A,B). Trypan blue staining revealed no pelotons in control protocorms (Supplementary Figure S1D), whereas abundant pelotons were observed in the basal cells of symbiotic protocorms (Supplementary Figure S1C). A colonization rate of 93.3 ± 3.33% confirmed successful colonization of *B. striata* protocorms by bj1.

**Figure 1:**
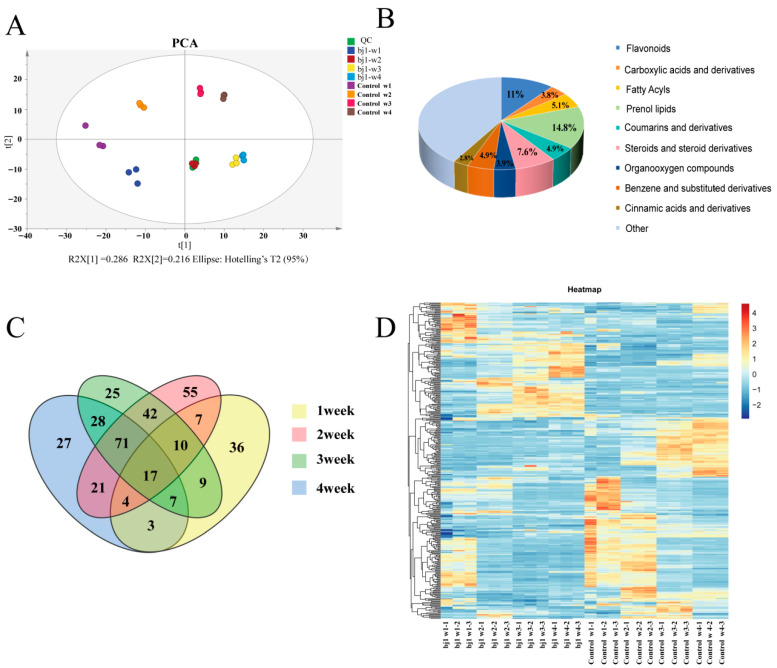
Metabolomic analysis. (**A**) the PCA score chart; (**B**) The compositional analysis of metabolites entails quantifying the relative abundance, expressed as a percentage value, of specific compound types within the overall pool of compounds; (**C**) Venn diagram of DAMs across weeks 1–4. (**D**) Heatmap of DAM relative abundances (blue: down-regulated; red: up-regulated; intensity reflects magnitude). Significant DAM differences between bj1 and control groups occurred at week 1. Moreover, the changes in DAMs at weeks 2, 3, and 4 differed from those observed at week 1, clearly indicating a pattern of regular changes.

The total ion chromatogram (Supplementary Figure S2) demonstrates excellent detection reproducibility, instrument stability, and chromatographic peak separation, meeting all requirements for subsequent analyses. Principal component analysis (PCA) revealed that principal component 1 (PC1) explained 28.6% of the total variance, while PC2 accounted for 21.6%, yielding a cumulative variance of 50.2% (Figure 1A). Notably, significant metabolic differences were observed across all time points, particularly between weeks 1 and 2. OPLS-DA modeling exhibited satisfactory fit (R^2^X, R^2^Y) and predictive (Q^2^) (Supplementary Table S2). The model showed highly significant separation between sample groups (Supplementary Figures S3A,B and S4A,B). Permutation testing confirmed model robustness with no evidence of overfitting (Supplementary Figures S3C–F and S4C–F).

A total of 526 compounds with known structures were identified, comprising 374 compounds in positive ion detection mode and 152 compounds in negative ion detection mode. Supplementary Table S3 provides comprehensive details on the identified metabolites, including compound name, *m*/*z* value, retention time, ionization mode, and molecular formula. The analysis reveals that the majority of these 526 metabolites can be categorized as follows: Prenol lipids (14.8%), Flavonoids (11%), Steroids and steroid derivatives (7.6%), Fatty Acyls (5.1%), Benzene and substituted derivatives (4.9%), Coumarins and derivatives (4.9%), Organooxygen compounds (3.9%), Carboxylic acids and derivatives (3.8%), Cinnamic acids and derivatives (2.8%) (Figure 1B).

DAMs were identified between bj1-treated and control groups across weeks 1–4, totaling 93, 227, 209, and 178 DAMs, respectively (Supplementary Figure S5). Specifically, upregulated/downregulated metabolites were: week 1 (50/43), week 2 (101/126), week 3 (79/130), and week 4 (76/102). The Venn Diagram was employed to determine the correlation among the DAMs (Figure 1C). The findings revealed that 17 metabolites were consistently present throughout all four stages of *B. striata* protocorms growth. Heatmap clustering analysis visualized relative abundances of differential metabolites in *B. striata* protocorms across 1–4 weeks (Figure 1D). The bj1 symbiotic treatment group showed highly significant metabolic changes at weeks 1–2, with trends toward consistency from week 2–4 (notably in weeks 3–4). The control group exhibited stable metabolite profiles at weeks 1–2, marked changes at week 2–3, and increasing consistency at week 3–4. Comparing the changes in DAMs across the two groups at various time points, it was observed that the number of differentials in the bj1 strain group consistently exhibited a decreasing trend. In contrast, the number of differentials in the control group initially increased before subsequently decreasing (Supplementary Figure S6). These observations suggest that under normal conditions, weeks 2 to 3 represent a critical phase of substantial metabolic reprogramming in *B. striata* protocorms. Meanwhile, weeks 1 to 2 mark an exceptionally dynamic period for the bj1 symbiotic treatment group, which aligns with the findings of the aforementioned PCA. The significant shifts in these metabolites imply underlying patterns of gene expression regulation and highlight the importance of weeks 1 to 2 as a pivotal stage for establishing the symbiotic relationship between the OM fungus bj1 and *B. striata* protocorms.

**Figure 2:**
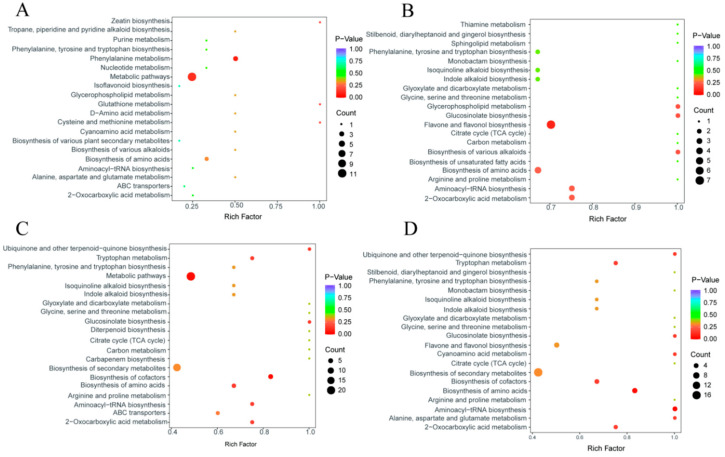
KEGG enrichment pathway analysis of DAMs. (**A**–**D**) was the KEGG enrichment map of differential metabolites at week 1, 2, 3 and 4, respectively; each bubble represents a metabolic pathway, with the size of the bubble indicating the proportion of compounds involved in KEGG enrichment analysis. The larger the bubble, the higher the proportion it represents. The top 20 most significantly enriched metabolic pathways were identified and ranked based on their *p*-values.

KEGG pathway analysis of significant DAMs using MetaboAnalyst (Version 6.0) (Figure 2) revealed predominant enrichment in metabolic pathways and biosynthesis of secondary metabolites. Notably, flavone and flavonol biosynthesis showed significant enrichment at weeks 2 and 4. These results demonstrate that bj1 strain critically impacts metabolic and secondary biosynthetic pathways during early growth of *B. striata* protocorms.

### 3.2. Sequencing and De Novo Assembly of B. striata Transcriptome and Functional Annotation

Based on metabolomic analysis, significant metabolite differences occurred between bj1-treated and control groups at weeks 1–2, with pronounced intra-group variations. RNA-seq was thus performed on these samples, yielding 66.21 Gb valid bases (89.74–94.71% valid rate) with high-quality metrics (Q20: 98.44–98.90%; Q30: 95.10–96.41%; GC: 47.14–48.79%; Supplementary Table S4). Pearson correlation and PCA confirmed excellent sample repeatability (high correlation coefficients, tight clustering; Figure S7), supporting subsequent analyses.

**Figure 3:**
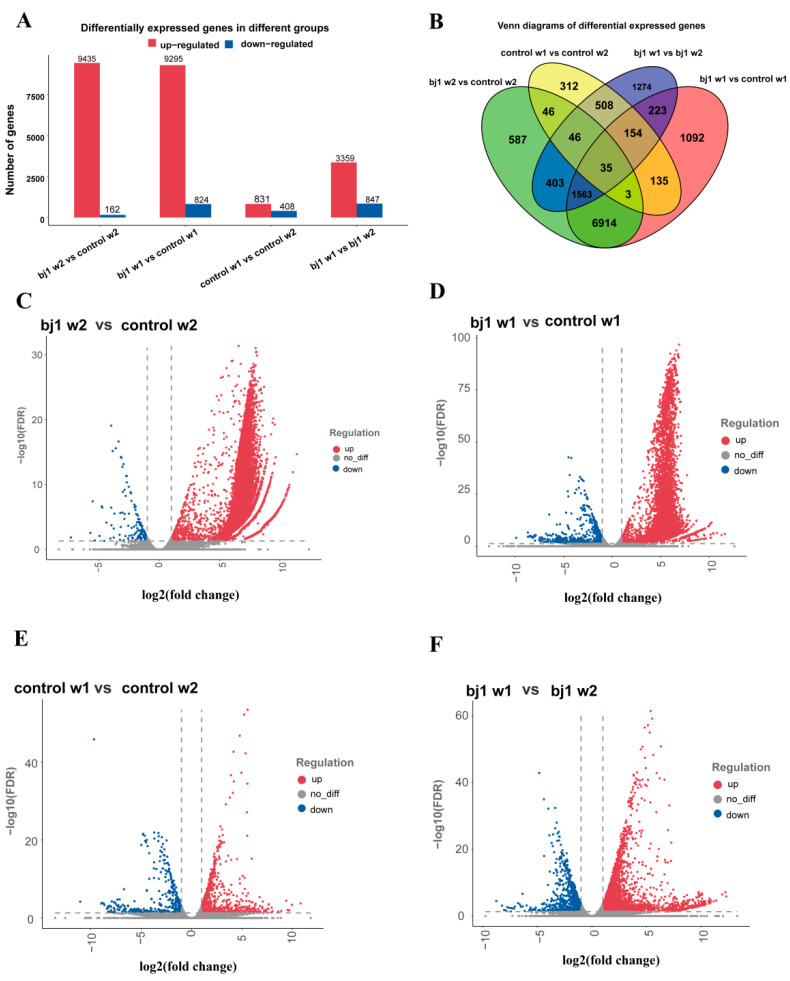
Transcriptome analysis. (**A**) Comparison of the number of genes between the bj1 group and the control group; (**B**) Venn diagram of differential genes; (**C**) Volcano map of DEGs in the (bj1 w2 vs. control w2) group; (**D**) Volcano map of DEGs in the (bj1 w1 vs. control w1) group; (**E**) Volcano map of DEGs in the (control w1 vs. control w2) group; (**F**) Volcano map of DEGs in the (bj1 w1 vs. bj1 w2) group.

The results showed a predominant positive correlation among most differential genes, with significantly more up-regulated genes (Figure 3A,B). In the (bj1 w1 vs. control w1) group, 10,119 genes were detected, including 9295 up-regulated and 824 down-regulated. The (bj1 w2 vs. control w2) group identified 9597 genes, with 9435 up-regulated and 162 down-regulated. The (control w1 vs. control w2) comparison revealed 831 up-regulated and 408 down-regulated genes (1239 total). In the (bj1 w1 vs. bj1 w2) analysis, 3359 genes were up-regulated and 847 down-regulated (4206 total) (Figure 3C–F). It also indirectly suggests that colonization by the OM fungi bj1 significantly enhances the expression levels of specific genes in *B. striata* protocorms, thereby promoting developmental processes.

A total of 92,942 gene sequences were successfully annotated. Differential expression gene (DEG) proportions in the four comparison groups were as follows: bj1 w2 vs. control w2 (10.32%), bj1 w1 vs. control w1 (10.89%), control w1 vs. control w2 (1.33%), and bj1 w1 vs. bj1 w2 (4.53%). Notably, DEG proportions were significantly higher in comparisons between bj1 treatment groups and their controls (bj1 vs. control) than in within-group time-point comparisons. This discrepancy is likely attributable to the fact that the bj1 treatment samples encompass two distinct transcriptomes: the *B. striata* protocorm transcriptome and the bj1 strain transcriptome. Consequently, all genes originating from the bj1 strain were classified as significantly up-regulated DEGs when compared with the control groups. However, due to the limitations of current sequencing technologies, it remains challenging to effectively filter genes without a reference genome. Thus, direct removal of bj1 strain transcripts was not feasible. To address this, we designated genes absent in both control timepoints but present in bj1 treatment groups as bj1 strain-specific genes and excluded them from downstream DEG analysis of symbiotic samples. Following this filtration, DEG comparisons between bj1 treatments and temporally matched controls (weeks 1 and 2) showed substantial decreases in total DEGs and putative up-/down-regulated genes (Supplementary Figure S8). Despite this reduction, overall expression trends remained consistent with pre-filtered results (Figure 3C,D).

**Figure 4:**
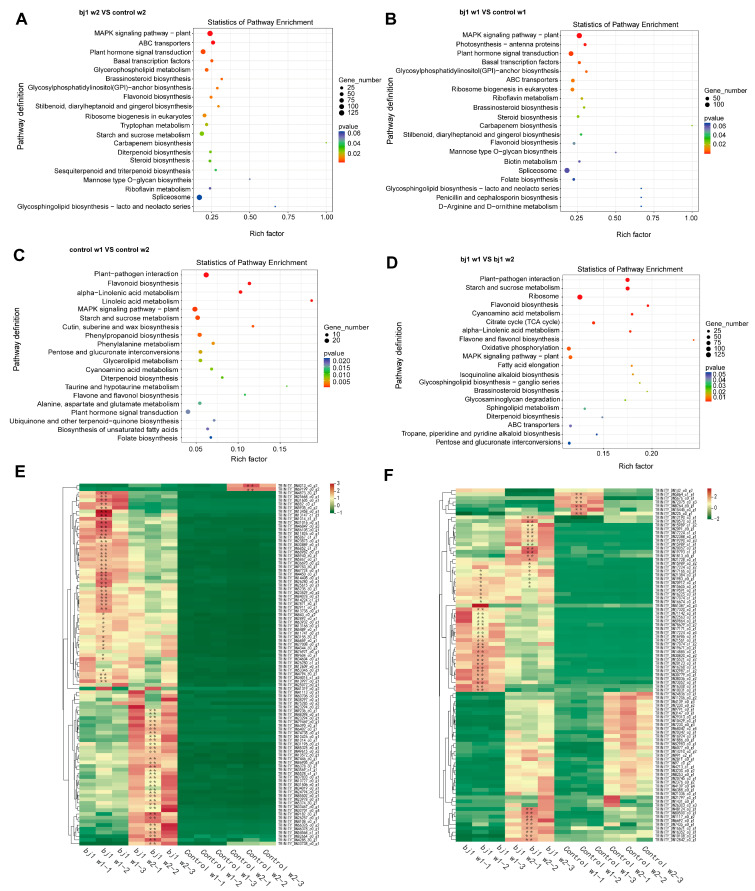
KEGG enrichment analysis of DEGs and heat map analysis of MAPK signaling pathway-plant related differential genes. (**A**) (bj1 w2 vs. control w2) group KEGG enrichment; (**B**) (bj1 w1 vs. control w1) group KEGG enrichment; (**C**) (control w1 vs. control w2) group KEGG enrichment; (**D**) (bj1 w1 vs. bj1 w2) group KEGG enrichment; The top 20 most significantly enriched metabolic pathways were identified and ranked based on their *p*-values; (**E**,**F**) heat map analysis of all MAPK signaling pathway-plant detected and associated DEGs; each color block in the heat map represents the relative expression of the gene at the corresponding location (green: down-regulated; red: up-regulated; intensity reflects magnitude). Most of the DEGs exhibited significantly higher expression levels in the symbiotic group compared to the control group. *: *p* < 0.05, **: *p* < 0.01.

GO enrichment analysis was conducted on DEGs in the samples (Supplementary Figure S9). The biological processes, nucleus, molecular functions, or protein binding exhibited the highest number of DEGs across the four groups. The DEGs in the sample were subjected to KEGG enrichment analysis, and the top 20 most significant metabolic pathways were identified (Figure 4A–D). The results demonstrate that the top three pathways of differentially enriched genes in group (bj1 w2 vs. control w2) are MAPK signaling pathway-plant, Spliceosome, and Plant hormone signal transduction, respectively. Similarly, the top three highest groups (bj1 w1 vs. control w1) exhibit consistent enrichment patterns as observed in the previous group. The comparison between control w1 and control w2 revealed Plant-pathogen interaction, MAPK signaling pathway-plant, and Starch and sucrose metabolism as the top three enriched pathways. In contrast, the top three enrichment pathways of differentially expressed genes in the comparison between bj1 w1 and bj1 w2 were Ribosome, Plant-pathogen interaction, and Starch and sucrose metabolism. Of the four groups mentioned above, three were primarily associated with the MAPK signaling pathway-plant, whereas the last two were both linked to starch and sucrose metabolism. Additionally, post-bj1 strain gene exclusion, KEGG analysis maintained significant enrichment of these critical pathways (Supplementary Figure S10). Consequently, the genes of the bj1 strain were no longer artificially removed in the subsequent data analysis.

**Figure 5:**
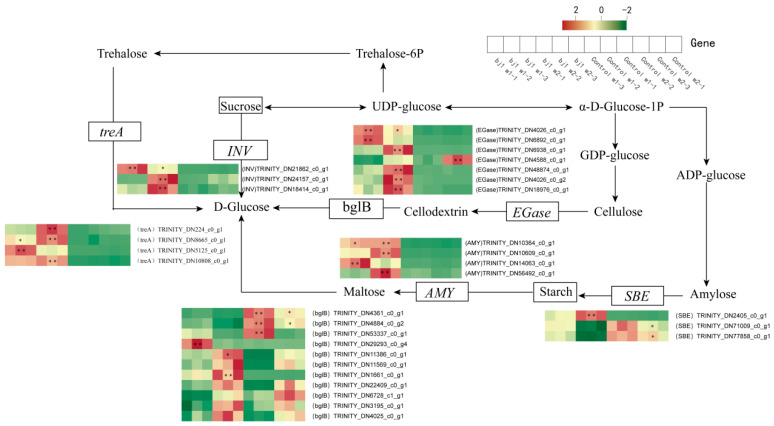
Symbiotic treatment with bj1 significantly promoted the expression of genes of starch and sucrose metabolic pathways in the protocorms of *B. striata*. Changes in gene expression of starch and sucrose metabolic pathways; The heat maps indicated relative expression levels of genes that encode the enzymes. *Egase*, endoglucanase; *bglB*, beta-glucosidase; *INV*: Invertase; *SBE*, 1,4-alpha-glucan branching enzyme; *AMY*, alpha-amylase; *treA*: α-trehalase. The independent sample t-test was employed to assess the statistical significance of differences between the two groups within the same week, *: *p* < 0.05, **: *p* < 0.01.

We compiled all differentially expressed genes identified in the MAPK signaling pathway-plant (Figure 4E,F) and Starch and sucrose metabolism pathways (Supplementary Figure S11) and compared their relative expression levels between the control and symbiotic groups. The results demonstrated that the expression levels of differentially expressed genes were significantly elevated in the symbiotic group for both pathways when compared to the control group, while most genes exhibited relatively lower expression levels in the control group. Although carbohydrate metabolites were not detected, considering the crucial role of the starch and sucrose metabolic pathways in seed germination and growth, we carried out an in-depth analysis of the changes in the expression levels of key genes within this metabolic pathway. The results indicated that several key hydrolase genes, including invertase (*INV*), endoglucanase (*EGase*), α-trehalase (*treA*), and α-amylase (*AMY*), exhibited significant symbiotic upregulation (Figure 5). qPCR validation confirmed these transcriptomic trends (Supplementary Figure S12), suggesting enhanced polysaccharide catabolism to glucose may improve carbohydrate utilization efficiency and promote protocorm development.

### 3.3. Dynamic Variations in Relative Contents of Flavonoid Metabolites

**Figure 6:**
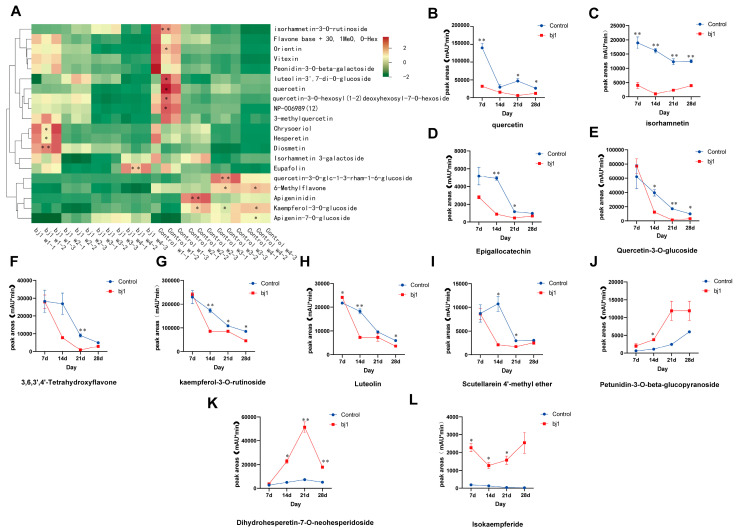
The relative contents of different flavonoids metabolites changed dynamically. (**A**) Heat map of relative flavonoid content; (**B**) Quercetin; (**C**) Isorhamnetin; (**D**) Epigallocatechin; (**E**) Quercetin-3-o-glucoside; (**F**) 3,6,3′,4′-tetrahydroxyflavone; (**G**) Kaempferol-3-o-rutinoside; (**H**) Luteolin; (**I**) Scutellarein 4′-methyl ether; (**J**) Petunidin-3-0-beta-glucopyranoside; (**K**) Dihydrohesperetin-7-0-neohesperidoside; (**L**) Isokaempferide; The independent sample *t*-test was employed to assess the statistical significance of differences between the two groups within the same week, *: *p* < 0.05, **: *p* < 0.01.

According to the statistical analysis of compound classification in metabolomics, flavonoids constituted a significantly substantial proportion among the total identified compounds (Figure 1B). However, the heat map analysis of flavonoid contents (Figure 6A) revealed a decreasing trend in the relative content of most flavonoids during *B. striata* seed germination and growth. This observation appears inconsistent with the expected effects on promoting plant growth and development, antioxidation, or facilitating seed germination. To further explore the potential biological effects of flavonoids on *B. striata* seed germination and growth, we analyzed their relative contents in *B. striata* protocorms under control and bj1 treatment conditions over four consecutive weeks. The results showed significant decreases in the levels of quercetin (Figure 6B), isorhamnetin (Figure 6C), epigallocatechin (Figure 6D), quercetin-3-o-glucoside (Figure 6E), 3,6,3′,4′-tetrahydroxy flavone (Figure 6F), kaempferol-3-O-rutinoside (Figure 6G), luteolin (Figure 6H), scutellarein-4′-methyl ether (Figure 6I) with the germination of *B. striata* seeds. Evidently, considering the natural germination of *B. striata* seeds in the control group, the reduction in these compounds implies that their high concentration is unfavorable for seed germination. Thus, it can be inferred that these compounds potentially act as germination inhibitors during *B. striata* seed germination. It is noteworthy that treatment with the bj1 strain resulted in a significant reduction in the relative contents of several flavonoids, including quercetin, isorhamnetin, and epigallocatechin, compared to the control group at week 1 (Figure 6B–D). In comparison to the control group, although no significant difference was observed in the relative content of other flavonoids at week 1 (Figure 6E–I), a significant decrease was noted at weeks 2 and 3. However, there was no significant difference between the two groups at week 4. These findings suggest that bj1 may expedite the degradation of aforementioned flavonoids, potentially alleviating their inhibitory effect on *B. striata* seed germination and thereby promoting their growth. Moreover, considering the broad-spectrum antibacterial activity exhibited by flavonoids, the overall reduction in their content may also facilitate the colonization of endomycorrhizal fungus bj1 and establishment of a symbiotic relationship with *B. striata* seeds, thereby promoting germination and growth.

Furthermore, our findings revealed a progressive increase in the relative content of petunidin-3-0-beta-glucopyranoside compound within the control group from week 1 to week 4 (Figure 6J). Similarly, this compound exhibited an upward trend from week 1 to week 3 in the bj1 group; however, it reached a plateau by week 4. The compound dihydrohesperetin-7-0-neohesperidoside exhibited a consistent linear increase from week 1 to week 3 in both the control and treatment groups, followed by a significant decrease at week 4 (Figure 6K). Notably, the relative contents of these two flavonoid compounds in the bj1 group were consistently higher than those observed in the control group after week 2. The compound isokaempferide exhibited a significant increase during the first week of bj1 infection, followed by a notable decrease in the second week. Subsequently, it showed another significant increase during the third and fourth weeks, while the control group demonstrated a gradual decline (Figure 6L). Whether these three flavonoids can promote the germination and growth of *B. striata* seeds needs further systematic study.

### 3.4. Analysis of Changes in Flavonoid Biosynthesis Based on Transcriptomics and Metabolomics

**Figure 7:**
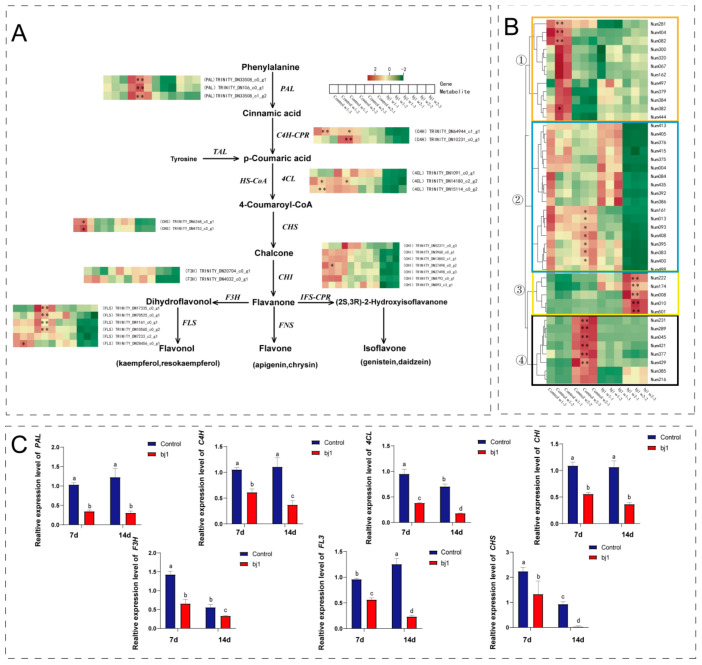
Symbiotic interaction with bj1 significantly suppressed the expression of genes associated with the flavonoid biosynthesis pathway in the protocorms of *B. striata*. (**A**) The flavonoid biosynthesis pathway and the heat map of relative expression of transcriptome genes; *PAL*: phenylalanine ammonia-lyase; *C4H*: cinnamate-4-hydroxylase; *TAL*: tyrosine ammonia-lyase; *4CL*: 4-coumarate:coenzyme A ligase; *CHS*: chalcone synthase; *CHI*: chalcone isomerase; *FNS*: flavone synthase; *F3H*: flavanone-3β-hydroxylase; *FLS*: flavanol synthase; *IFS*: isoflavone synthase; *CPR*: cytochrome P450 reductase; (**B**) The heat map of relative metabolite content, *: *p* < 0.05, **: *p* < 0.01; (**C**) qPCR validation of flavonoid biosynthetic gene expression in *B. striata* protocorms demonstrated time-dependent and treatment-specific variations. Statistical significance (*p* < 0.05) between groups marked with distinct superscript letters was confirmed through two-way ANOVA with Tukey’s post hoc test.

Correlation analysis was conducted to elucidate the dynamic relationship between the identified flavonoids in metabolomics and genes associated with the biosynthetic pathway of flavonoids as demonstrated in Figure 7A [26,27,28]. Based on the heat map analysis of relative gene expression levels within the transcriptome, the results indicated that, in comparison to the control group, the symbiosis group demonstrated a downregulation in the expression levels of enzymes involved in flavonoid biosynthesis, including phenylalanine ammonia-lyase (*PAL*), CYP450-based cinnamate-4-hydroxylase (*C4H*), coenzyme A ligase (*4CL*), chalcone synthase (*CHS*), chalcone isomerase (*CHI*), flavanone-3β-hydroxylase (*F3H*) and flavonol synthase (*FLS*) (Figure 7A). Furthermore, a reduction in flavonoid content was also observed. The flavonoids were categorized into four groups (Figure 7B): In group 1, the control compound exhibited high content in the first week, which significantly declined in the second week; conversely, the symbiosis group consistently displayed low content throughout both weeks. In group 2, both the control and symbiosis groups demonstrated high expression levels during the initial week. While this expression was predominantly sustained in the control group during the second week, it decreased in the symbiosis group. Group 3 witnessed consistently lower content in the control group compared to a significant increase observed in the symbiosis group during Week 2. Lastly, while there was a notable increase observed within the control group during Week 2 of Group 4, minimal overall change occurred within its counterpart the symbiosis group. In general, after symbiosis with the bj1 strain, the flavonoid content in group 3 increased, whereas the flavonoid content in other groups decreased. Moreover, the expression levels of flavonoid biosynthesis genes in the two groups were verified using qPCR. The results demonstrated a significant down-regulation of related genes involved in flavonoid biosynthesis, including *PAL*, *C4H*, *4CL*, *CHS*, *CHI*, *F3H* and *FLS*, after treatment with the bj1 strain compared to the control group. These findings are consistent with the transcriptome analysis results mentioned above (Figure 7A).

### 3.5. Analysis of Changes in IAA Biosynthesis Based on Transcriptomics and Metabolomics

**Figure 8:**
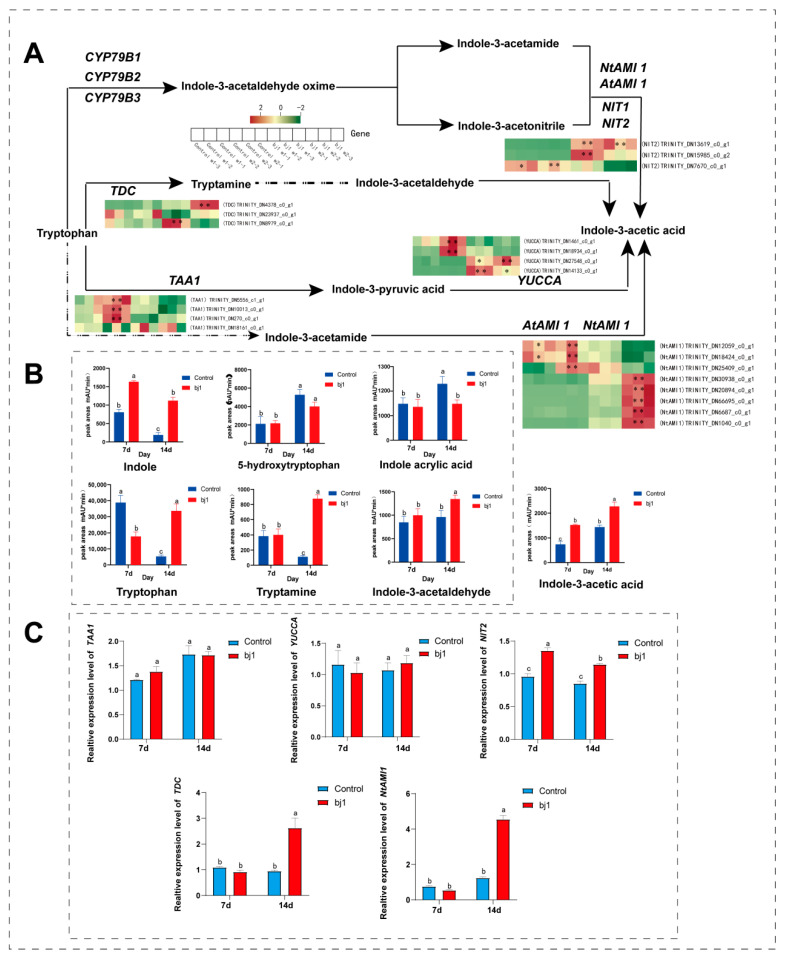
Symbiotic interaction with bj1 significantly enhanced the expression of genes associated with the IAA biosynthesis pathway in the protocorms of *B. striata* and increased the accumulation of IAA. (**A**) IAA biosynthetic pathway and the heat map of relative expression of transcriptome genes; *TAA1*: Tryptophan Amino Transferase of Arabidopsis; *TDC*: tryptophan decarboxylase; *NtAMI*: acetamide hydrolase; *YUCCA*: flavin monooxygenase-like enzyme; *NIT2*: omega-amidase, *: *p* < 0.05, **: *p* < 0.01; (**B**) The relative peak area of metabolome compounds; (**C**) qPCR verification of the expression levels of IAA biosynthesis genes in *B. striata* protocorms. Statistical significance (*p* < 0.05) between groups marked with distinct superscript letters was confirmed through two-way ANOVA with Tukey’s post hoc test.

During early stages of development, numerous plants autonomously synthesize auxin via both tryptophan-dependent and non-tryptophan-dependent metabolic pathways as depicted in Figure 8A [29,30]. Combined analysis of IAA synthesis-related gene enzymes and IAA synthesizing compounds and derivatives showed that: A total of 5 enzymes related to IAA synthesis were detected, including tryptophan decarboxylase (*TDC*), Tryptophan Amino Transferase of Arabidopsis (*TAA1*), flavin monooxygenase-like enzyme (*YUCCA*), omega-amidase (*NIT2*) and acetamide hydrolase (*NtAMII*) in the symbiotic group, the expression levels of *TDC*, *YUCCA*, *NIT2*, and *NtAMI* were significantly higher compared to those in the control group, exhibiting a pronounced up-regulated trend. Conversely, the expression levels of TAA1 were lower in the symbiotic group than in the control group and displayed a down-regulated trend (Figure 8A).

In conjunction with the observed IAA-related compounds and derivatives in the metabolic group, a significant increase in relative IAA content was found with prolonged culture time. The symbiotic group exhibited higher levels compared to the control group. Additionally, there was an elevation in tryptamine compounds and indole-3-acetaldehyde within the tryptamine pathway. Furthermore, the relative content of indole compounds in the symbiotic group surpassed that of the control group; however, both groups displayed a decrease over time. The relative concentrations of indole acrylic acid and 5-hydroxytryptophan were found to be higher in the control group compared to the symbiotic group, while the levels of tryptophan compounds exhibited an initial increase followed by a subsequent decrease in the control group, whereas the opposite trend was observed in the symbiotic group (Figure 8B). In conclusion, strain bj1 may enhance the biosynthesis of IAA by regulating the upregulation of *TDC* in the tryptophan pathway and *NtAMI* in the indole acetamide pathway, thereby jointly promoting *B. striata* seed germination. The gene expression levels in the two groups of IAA biosynthesis pathways were determined using qPCR. The results revealed a significant increase in the expression levels of *TDC*, *NtAMI*, and *NIT2* in the symbiotic group compared to the control group. Conversely, the expression levels of *TAA1* and *YUCCA* were decreased, although no significant difference was observed between the two groups (Figure 8C). These findings from qPCR analysis are consistent with the transcriptome changes mentioned above.

### 3.6. Analysis of Changes in Jasmonic Acid Biosynthesis Based on Transcriptomics and Metabolomics

**Figure 9:**
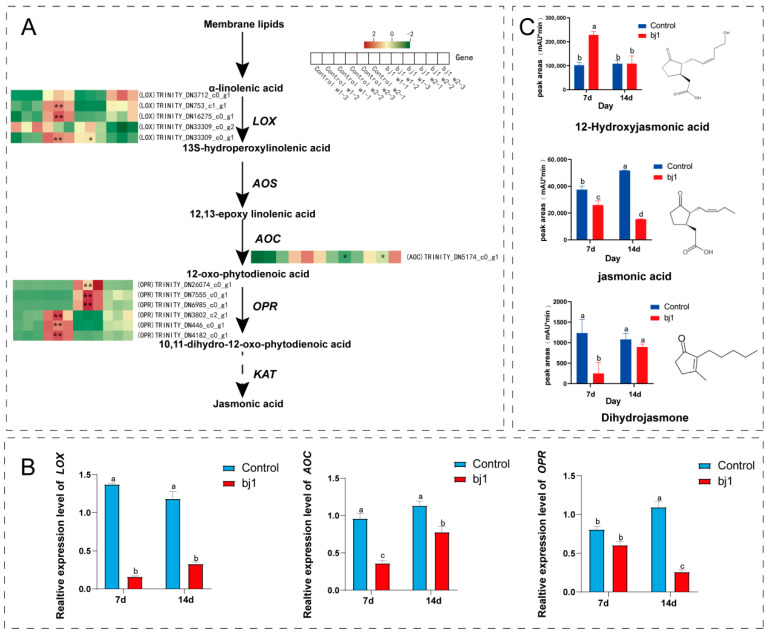
The symbiotic interaction between bj1 and *B. striata* significantly downregulated the expression of genes associated with the JA biosynthesis pathway in *B. striata* protocorms, while promoting the accumulation of 12-hydroxyjasmonic acid. (**A**) JA biosynthetic pathway and the heat map of relative expression of transcriptome genes; *LOX*: lipoxygenase; *AOS*: allene oxide synthase; *AOC*: allene oxide cyclase; *OPR*: 12-oxophytodienoate reductase; *KAT*: 3-ketoacyl-CoA thiolase, *: *p* < 0.05, **: *p* < 0.01; (**B**) qPCR verification of the expression levels of flavonoid biosynthesis and IAA biosynthesis genes in *B. striata* protocorms; (**C**) The relative peak area of metabolome compounds. Statistical significance (*p* < 0.05) between groups marked with distinct superscript letters was confirmed through two-way ANOVA with Tukey’s post hoc test.

We analyzed correlation between key jasmonate synthesis-related enzymes and jasmonate derivatives based on the established jasmonate pathway as depicted in Figure 9A [31,32]. Transcriptomic revealed downregulation of jasmonate biosynthetic enzymes, including lipoxygenase (*LOX*), allene oxide cyclase (*AOC*) and 12-oxophytodienoate reductase (*OPR*), in bj1-treated protocorms versus control. qPCR confirmed significant suppression of these genes (Figure 9B). Consistently, JA levels were substantially reduced in the bj1 group (Figure 9C).

Intriguingly, in addition to JA, two JA derivatives, namely dihydrojasmone and 12-hydroxyjasmonic acid were detected. The former decreased significantly in bj1-treated protocorms at week 1 but rebounded by week 2. Conversely, the latter increased markedly at week 1 with no significant difference versus controls at week 2 (Figure 9C). This metabolic shift likely reflects both reduced JA biosynthesis and enhanced conversion to 12-hydroxy-jasmonic acid (12-OH-JA) following bj1 colonization. Given the established roles of JA, dihydrojasmone and JA in plant immune regulation [33,34], the observed alterations in these signaling molecules may suppress immune response in *B. striata* protocorms. This modulation could facilitate bj1 invasion, colonization, and ultimately promote mutualistic establishment.

### 3.7. Correlation Network Analysis of Gene Expression and Metabolites

**Figure 10:**
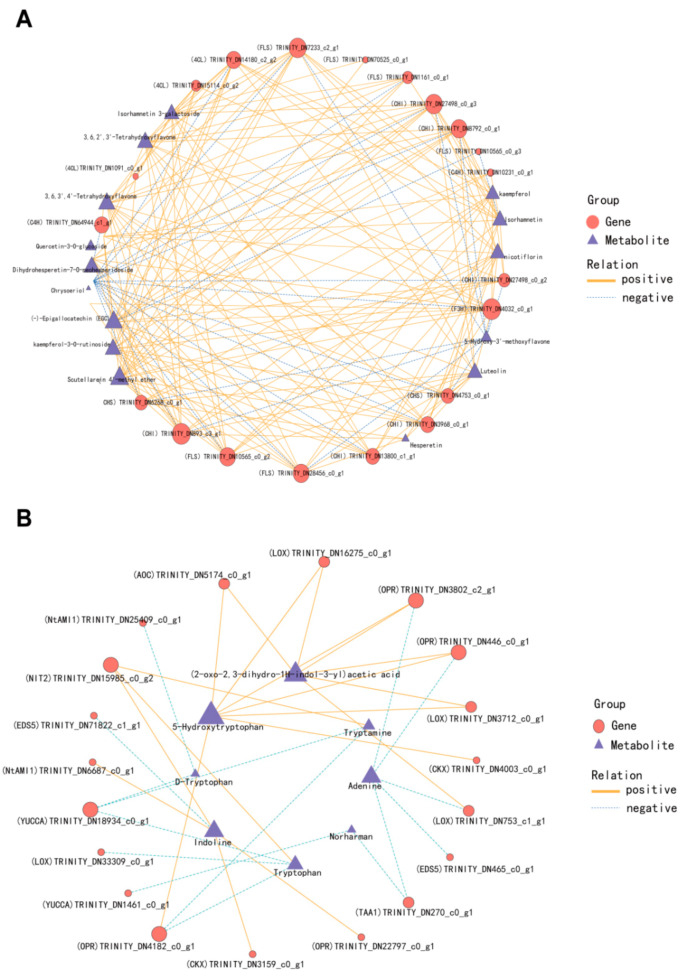
Correlation network analysis of DEGs and DAMs in the biosynthesis pathway of flavonoids and plant hormones. (**A**) Flavonoid biosynthesis pathway; (**B**) Plant hormone biosynthesis pathway. The red circle represents genes, the dark blue triangle signifies metabolites, the solid yellow line denotes a positive correlation between the two, and the light blue dashed line indicates a negative correlation.

In the pathway of flavonoid biosynthesis, the two genes (*CHI*) TRINITY_DN893_c3_g1 and (*F3H*) TRINITY_DN4032_c0_g1 had the most linked metabolites. It was negatively correlated with 5-Hydroxy-3′-methoxyflavone and dihydrohesperetin-7-O- neohesperidoside, and positively correlated with other flavonoids. Scutellarein 4′-methyl ether has the largest number of genes linked by metabolites, including *C4H*, *4CL*, *CHI*, *FLS* and *F3H.* In the plant hormone synthesis pathway, 5-hydroxytryptophan had the largest number of linked genes, which were positively correlated with *LOX*, *AOC* and *OPR*, while adenine was negatively correlated with *TAA1*, *LOX* and *OPR* (Figure 10).

## 4. Discussion

Seed germination and early seedling development in plants are regulated by complex interactions between environmental factors (light, temperature) and biological components (biotic/abiotic elements) [35]. Orchidaceae species exhibit unique germination characteristics due to their dust-like seeds lacking endosperm and obligate dependence on mycorrhizal fungi for nutrient acquisition [36]. Substantial evidence demonstrates that orchid mycorrhizal associations enhance seed metabolic processes through active participation in transcriptional regulation and signal transduction during symbiotic germination [1], resulting in significant alterations in plant energy metabolism and defense-related gene expression [37]. Proteomic analyses of *Oncidium sphacelatum* revealed fungal-induced reprogramming of carbon metabolism pathways coupled with increased accumulation of ROS homeostasis regulators, phytoalexins, and carotenoid biosynthesis proteins [38]. Complementary transcriptomic studies in *Dendrobium* identified symbiotic germination-associated genes involved in stress response, metabolic regulation, and signal transduction [39]. However, the molecular mechanisms underlying *B. striata* seed germination mediated by *Tulasnella* sp. strain bj1 remain poorly characterized.

To elucidate these mechanisms, we conducted integrated transcriptomic and metabolomic analyses. Metabolite profiling identified 526 compounds, with prenol lipids (14.8%) and flavonoids (11%) constituting the dominant classes. Multivariate analysis (PCA/OPLS-DA) revealed significant metabolic divergence between symbiotic and asymbiotic groups, particularly during early germination (1–2 weeks). This temporal specificity likely reflects the developmental status of *B. striata* protocorms: incomplete organogenesis during weeks 1–2 necessitates fungal metabolic support, whereas week 3–4 samples with mature organs showed metabolic stabilization. Morphological observations aligned with these findings—seed coat disintegration initiated at 6–7 days, cotyledon differentiation by day 14, and transition to autotrophic seedlings by week 3–4 [20]. These results underscore the critical importance of early-stage molecular investigations for understanding symbiotic establishment.

Transcriptome analysis revealed that the majority of DEGs were positively associated with seed development and bj1 symbiosis. In the comparisons of (bj1 w1 vs. control w1), (bj1 w2 vs. control w2), and (bj1 w1 vs. bj1 w2), a significant upregulation of DEGs was observed. Conversely, in the (control w1 vs. control w2) group, the number of significantly upregulated DEGs was considerably lower than in the aforementioned three groups. Transcriptomic studies have demonstrated that the proportion of DEGs varies substantially across species, experimental designs, comparison conditions, and analytical methods [40]. Notably, we observed that over 9000 genes were significantly upregulated in the bj1-treated samples compared to the control group, whereas only approximately 1200 genes exhibited significant changes between the first and second weeks in the control group. Similarly, Miura et al. analyzed the transcriptome of *B. striata* seeds germinating symbiotically with *Tulasnella* sp. [6]. HR1-1 and found that more than 6000 DEGs were detected in both the first and second weeks when comparing symbiotic and non-symbiotic treatments. We speculate that this discrepancy may primarily arise from the presence of two distinct transcriptomes in bj1-treated samples: the *B. striata* protocorm transcriptome and the bj1 strain transcriptome. To date, no data analysis or processing methods for filtering out the genes of a specific species have been reported in similar studies due to technical limitations [1,6,37]. To address this issue, we attempted to manually remove potential bj1-related genes and reanalyze the DEGs between the bj1-treated and control groups. The results indicated that while the number of DEGs decreased significantly (Supplementary Figure S8), the enriched pathways (Supplementary Figure S10) remained largely consistent with those identified prior to removal. Furthermore, validation of key genes involved in sugar, flavonoid, and hormone-related pathways confirmed that these genes were predominantly expressed by *B. striata* protocorms and effectively reflected the expected expression patterns during symbiotic germination. Literature reports suggest that fungal colonization typically induces increased gene activity in plants. In contrast, symbiotic mycorrhizal fungi often exhibit more negatively regulated gene expression or may become inactive [41]. Based on these findings, the potential interference of bj1 genes in the sequencing results will not be considered in subsequent data analysis and interpretation.

The starch and sucrose metabolic pathways play a critical role in the germination and growth of plant seeds. KEGG enrichment analysis and qPCR validation revealed that symbiotic treatment with bj1 significantly upregulated the expression of polysaccharide-degrading genes, including *INV*, *EGase*, *treA*, and *AMY*, within this pathway (Figure 5). This upregulation promotes the degradation of starch granules in seeds [42] and enhances the digestion of bj1 pelotons [4], thereby providing essential energy for the growth and development of *B. striata* protocorms. Recent studies have shown that mycorrhizal fungi can provide water-soluble trehalose to orchid seeds. Thus, the expression of trehalose hydrolase (e.g., *treA*) is significantly induced during symbiotic germination [3], and our results are consistent with those reported in the literature. Interestingly, different orchid mycorrhizal fungi exhibit variations in their ability to utilize carbon sources. Mehra et al. found that mycorrhizal fungi incapable of utilizing sucrose lack sucrose transporters or active sucrose-decomposing enzymes, such as *INV* [43]. This prevents them from competing with host plants for sucrose, thereby promoting the growth of orchid plants in later developmental stages. In contrast, mycorrhizal fungi capable of utilizing sucrose may cause a shift from symbiotic relationships to parasitic ones. In conclusion, OM fungi may influence their symbiotic relationships with orchids through carbon source utilization—a topic that warrants further in-depth investigation.

Flavonoids are synthesized, transported, and secreted in plants with conserved properties that facilitate interactions with microorganisms and play pivotal roles. As essential antibacterial compounds, reduced flavonoid levels increase plant susceptibility to pathogen infection and microbial invasion [44,45]. For instance, knockdown of the transcription factor HvWRKY23 in barley downregulated key flavonoid biosynthesis genes, decreased flavonoid accumulation, and ultimately enhanced *Fusarium graminearum* infection [45]. Notably, infection by the bj1 strain significantly reduced flavonoid abundance in *B. striata* protocorms, potentially facilitating microbial colonization and symbiotic establishment. While certain flavonoids and terpenoids promote mycorrhizal colonization–exemplified by quercetin-mediated arbuscular mycorrhizal promotion in *Triadica sebifera* roots [46], and strigolactone-facilitated mucor colonization in *Arabidopsis* [47]. We noticed that bj1 colonization specifically increased petunidin-3-0-beta-glucopyranoside, dihydrohesperetin-7-0-neohesperidoside, and isokaempferide levels in *B. striata*. These compounds require further investigation to assess their signaling similarity and functional roles during infection. Furthermore, flavonoids have been found to inhibit seed germination and growth under certain circumstances by inhibiting the secretion of endogenous plant hormones such as auxins, gibberellins, and cytokinins [48]. It was found that fungal inoculation could activate the flavonoid biosynthesis pathway in orchid tubers while inhibiting auxin metabolism and transport [37]. The relative flavonoid contents in *B. striata* protocorms significantly decreased with seed germination and growth in the control group. Evidently, this decline in flavonoid content positively influenced the germination and growth of *B. striata* seeds. Therefore, we hypothesize that the accumulation of flavonoids in *B. striata* seeds may contribute to seed dormancy rather than germination.

A comprehensive analysis of the transcriptomic and metabolomic data revealed that bj1 infection significantly modified the gene expression profile of *B. striata* protocorms. This led to a significant down regulation of genes involved in flavonoid synthesis pathways, accompanied by a notable reduction in the content of flavonoid components. Previous research has demonstrated that decreased expression of key genes, including *PAL*, *FLS*, *C4H*, *4CL*, *CHS*, and *CHI*, correlates with a decline in the levels of flavonoid compounds such as quercetin, isorhamnetin, epigallocatechin, and quercetin-3-O-glucoside [49]. The results of this study are highly congruent with these findings. Notably, *CHS* expression in bj1-symbiotic protocorms declined markedly during Week 1 and became nearly undetectable by Week 2 (Figure 7C). As *CHS* encodes the initial rate-limiting enzyme in plant flavonoid biosynthesis, its suppression directly impedes accumulation of key secondary metabolites including flavones, flavonols, isoflavones, and anthocyanins [50]. Overall, these results offer mechanistic insights into the flavonoid depletion induced by bj1 infection.

Plant hormones orchestrate plant developmental processes and environmental responses. Studies demonstrate that mycorrhizal fungi can produce auxins to accelerate host growth and development, a capability confirmed in our preliminary work with the bj1 strain [22]. In the current work, integrated multi-omics analysis revealed upregulation of IAA biosynthetic enzymes in symbiotic groups, particularly at week 2, accompanied by significantly elevated intermediate metabolites in the IAA pathway compared to controls. These findings align with both enzymatic expression patterns and qPCR validation, demonstrating that bj1 symbiosis enhances IAA production through upregulated *TDC* (tryptophan pathway) and *NtAMI* (indole acetamide pathway), thereby jointly promoting *B. striata* seed germination and growth. However, further investigation is required to determine whether the interaction between bj1 and *B. striata* seeds promotes the expression of seed-related genes or if the expression of bj1 strain-related genes leads to the accumulation of metabolites related to the IAA pathway, as bj1 [22] and numerous other endophytic fungi are competent in synthesizing IAA [51,52].

JA is an essential plant hormone in regulating plant growth, with its primary physiological role being the activation of plants’ immune responses to combat pathogenic microorganisms. However, excessive levels of JA components may hinder symbiotic interactions with microorganisms [53]. In the symbiotic system between *Dendrobium* seeds and *Tulasnella* sp. fungi, Wang et al. observed significant down-regulation of jasmonate-related synthetase expression in symbiotic versus asymbiotic groups [27]. This transcriptional suppression was postulated to result from fungal colonization-mediated inhibition of JA biosynthetic genes, thereby facilitating fungal entry into cortex cells and establishing stable mutualistic symbiosis. Consistently, our investigation revealed analogous downregulation patterns in both JA pathway-related genes and their metabolites within bj1 (*Tulasnella* sp.)-infected *B. striata* protocorms (Figure 9C). Additionally, relevant studies have also revealed that fungi secrete an antibiotic biosynthesis monooxygenase (*Abm*) into plant cells during host plant infection, thereby participating in the regulation of plant immune response. However, this *Abm* enzyme has the ability to convert JA into 12-OH-JA, which inhibits the immune response of plants and facilitates fungal infection and dissemination within host plants [33]. Further investigations are required to determine whether the bj1 strain possesses the same Abm gene.

Although this study establishes a macro framework by which bj1 co-regulates JA, flavonoid, and IAA pathways to facilitate symbiotic germination, several precise molecular mechanisms remain elusive. Future investigations should address the following key questions: (1) Genetic basis of JA conversion: Is the conversion of JA to 12-OH-JA driven by specific enzymes encoded in the bj1 genome or by bj1-induced host seed genes? (2) Causal role of JA signaling changes: Which factor is more critical for bj1 colonization and symbiosis—the reduction in JA levels, accumulation of 12-OH-JA, or their synergistic effect? (3) Regulatory pathways of flavonoid metabolism: Beyond suppressing host flavonoid biosynthetic genes, does bj1 possess or secrete functional genes that promote flavonoid transformation or degradation? (4) Ecological function of flavonoids: Do flavonoid monomers with significantly decreased content (e.g., quercetin, isorhamnetin) indeed inhibit bj1 growth and infection? To resolve these questions, subsequent work should focus on: (1) Mining and functionally validating key genes via bj1 transcriptome/genome analysis to identify JA hydroxylation candidates, with verification through gene knockout/complementation in symbiotic systems; (2) Conducting exogenous application experiments (JA, 12-OH-JA, or pathway inhibitors) in symbiotic systems to systematically evaluate their independent and combined effects on bj1 colonization rate, peloton formation, host immune marker gene expression, and seed germination; (3) Elucidating the cross-kingdom flavonoid metabolic network via integrated analysis of flavonoid metabolism/degradation genes in both bj1 and *B. striata* seeds, with functional validation through heterologous expression or enzyme activity assays; (4) Directly testing the antifungal activity of flavonoids in vitro to determine the inhibitory effects of representative monomers on bj1 hyphal growth, spore germination, and infection structure formation; (5) Achieving spatiotemporal localization via in situ hybridization, immunohistochemistry, or spatial metabolomics to map the expression of JA conversion/flavonoid degradation genes and the accumulation of corresponding metabolites—particularly at fungal entry sites—to reveal microchemical dynamics at the interaction interface. A systematic dissection of these mechanisms will not only complete the molecular map of the bj1-*B. striata* seed interaction but also hold significant translational promise. Ultimately, this work will establish a solid scientific foundation for precisely manipulating the *B. striata*-fungus symbiotic system.

Given the medicinal value of *B. striata* and the industrial potential of symbiotic germination, the practical limitations and scalability of bj1-based inoculation warrant thorough evaluation. Although our study shows that bj1 significantly enhances seed germination and protocorm development, several critical challenges must be overcome to enable nursery-scale application, along with targeted optimization strategies: (1) To support large-scale production of the strain, it is essential to establish a robust quality control system. By employing techniques such as liquid fermentation, mycelial yield can be significantly enhanced while ensuring the viability of the mycelium and the sustained activity of functional enzymes. In addition, it is essential to mitigate contamination risks, refine the seed disinfection protocol, and achieve a balanced colonization of beneficial microbes while suppressing harmful microorganisms. (2) During the seed harvesting process, it is essential to establish clear standards for seed viability, adopt production-scale operational protocols, align with conventional nursery conditions, and offset inoculation costs by enhancing germination rates and seedling quality. In summary, through process optimization and standardized management, bj1 exhibits substantial application potential as a symbiotic inoculant in *B. striata* nursery production, offering technical support for the sustainable development of the industry.

## 5. Conclusions

**Figure 11:**
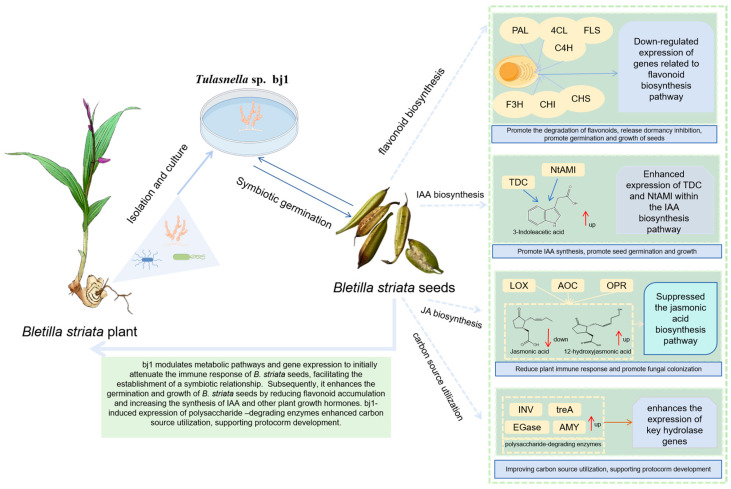
Molecular mechanism of symbiotic germination of *B. striata* seeds (created with Figdraw.com).

In conclusion, the current study provides novel integrated transcriptomic and metabolomic insights demonstrating that the specific mycorrhizal fungus bj1 modulates key metabolic pathways in *B. striata* to facilitate symbiotic germination. Specifically, the bj1 significantly suppresses the expression of genes involved in JA biosynthesis in *B. striata*, with a concurrent reduction in JA levels and accumulation of 12-OH-JA—changes that may collectively reduce the immune response of *B. striata* seeds to exogenous microorganisms and favor the establishment of a symbiotic germination system. Furthermore, bj1 potentially down-regulates genes associated with the flavonoid biosynthesis pathway. The resultant transcriptional repression coupled with decreased flavonoid relative abundance suggests that dampening flavonoid synthesis or promoting its reduced accumulation is a regulatory strategy contributing to *B. striata* seed germination and growth—an underappreciated link between flavonoid metabolism and orchid-mycorrhizal symbiosis. Lastly, bj1 notably up-regulates TDC expression in the tryptophan pathway and NtAMI expression in the indole acetamide pathway to enhance IAA synthesis, while simultaneously inducing polysaccharide-degrading enzyme expression to improve carbon source utilization and support protocorm development. Collectively, bj1 synergistically promotes *B. striata* seed germination and growth by orchestrating plant immune response, flavonoid metabolism, hormone synthesis, and carbon source utilization (Figure 11), which advances our understanding of the molecular crosstalk underlying orchid symbiotic germination with broader implications for plant-microbe mutualism.

## Data Availability

The original contributions presented in the study are included in the article/Supplementary Material, further inquiries can be directed to the corresponding authors.

## Supplementary Materials

The following supporting information can be downloaded at: https://www.mdpi.com/article/10.3390/microorganisms14010174/s1, Figure S1: Germination of *B. striata* protocorms and colonization by the mycorrhizal fungal strain bj1. A: *B. striata* protocorms grown symbiotically with bj1 (bar = 1 cm); B: *B. striata* protocorms grown asymbiotically (bar = 1 cm); C: Intracellular colonization by bj1 within a *B. striata* protocorm (arrow) (bar = 50 μm); D: Control *B. striata* protocorm without bj1 colonization (bar = 50 μm); Figure S2: Total ion current in *B. striata*; A: the total ion current diagram of positive ion mode; B: the total ion current diagram of the negative ion mode; Figure S3: OPLS-DA, displacement test and S-Plot of samples from the bj1 group and control group at 1 week and 2 weeks. A: The OPLS-DA score chart of the 1 week; B: The OPLS-DA score chart of the 2 weeks; C: The displacement test chart of the 1 week; D: The displacement test chart of the 2 weeks; E: The S-Plot of the 1 week; F: The S-Plot of the 2 weeks; Figure S4: OPLS-DA, displacement test and S-Plot of samples from the bj1 group and control group at 3 weeks and 4 weeks; A: The OPLS-DA score chart of the 3 weeks; B: The OPLS-DA score chart of the 4 weeks; C: The displacement test chart of the 3 weeks; D: The displacement test chart of the 4 weeks; E: The S-Plot of the 3 weeks; F: The S-Plot of the 4 weeks; Figure S5: Volcanic map analysis of different metabolites between bj1 group and control group; A-D were the differential metabolite volcano maps of week 1, week 2, week 3 and week 4, respectively. Each point in the volcano map represents a metabolite, the abscissa represents the quantitative difference in the two samples multiplied by the logarithm of the metabolite, and the ordinate represents the level of significance of the differential metabolite. The green/red dots in the figure indicate down-regulated/up-regulated differential metabolites, respectively; Figure S6: Changes in DAMs at different time points in the bj1 and control groups; Figure S7: Pearson correlation coefficient and PCA diagram; A: Pearson correlation coefficient. The horizontal and vertical coordinates are for each sample, respectively, and the color depth indicates the correlation coefficient size of the two samples. The closer it is to red (the closer the coefficient is to 1) the greater the correlation; the closer it is to white, the less correlated it is; B: PCA principal component analysis diagram; Figure S8: Transcriptome analysis of *B. striata* seeds after exclusion of genes from the transcriptome of bj1 strain. A: Volcano map of DEGs in the (bj1 w1 vs. ctrl w1) group; B: Volcano map of DEGs in the (bj1 w2 vs. ctrl w2) group; Figure S9: GO enrichment analysis of differential genes between the bj1 group and the control group. A: (bj1 w1 vs. ctrl w1) group GO enrichment; B: (bj1 w2 vs. ctrl w2) group GO enrichment; C: (bj1 w1 vs. bj1 w2) group GO enrichment; D: (ctrl w1 vs. ctrl w2) group GO enrichment; Figure S10: KEGG analysis of *B. striata* seeds after exclusion of genes from the transcriptome of bj1 strain; Figure S11: Heat map analysis of all Starch and sucrose metabolism detected and associated DEGs; each color block in the heat map represents the relative expression of the gene at the corresponding location, and the green/red block in the figure represents the down-regulated/ up-regulated differential genes respectively. The darker the color, the higher the content, and vice versa. Most of the DEGs exhibited significantly higher expression levels in the symbiotic group compared to the control group. The independent sample t-test was employed to assess the statistical significance of differences between the two groups within the same week, *: *p* < 0.05, **: *p* < 0.01; Figure S12: qPCR verification of the expression levels of starch and sucrose metabolic pathways genes in *B. striata* protocorms. Significant differences between groups are indicated by different letters (a, b, c and d), *p* < 0.05, by two-way ANOVA with Tukey’s post hoc test; Table S1: *B. striata* genes and primers; Table S2: Cumulative interpretation rate of OPLS-DA model between samples; Table S3: Summary of Identified Metabolites in *B. striata* protocorms; Table S4: Statistical table of raw transcriptome data of *B. striata* protocorms at 1–2 weeks.
