# Supplementary material for: Deciphering Molecular Pathways of Bletilla striata Seeds Symbiotic Germination with Tulasnella sp. bj1
Source: Microorganisms. 2026 Jan 13;14(1):174. doi: 10.3390/microorganisms14010174 (PMC12844467; doi:10.3390/microorganisms14010174)
Supplement: Supplementary file 1 [file microorganisms-14-00174-s001.zip › Supplementary Fig 1.1.pdf]

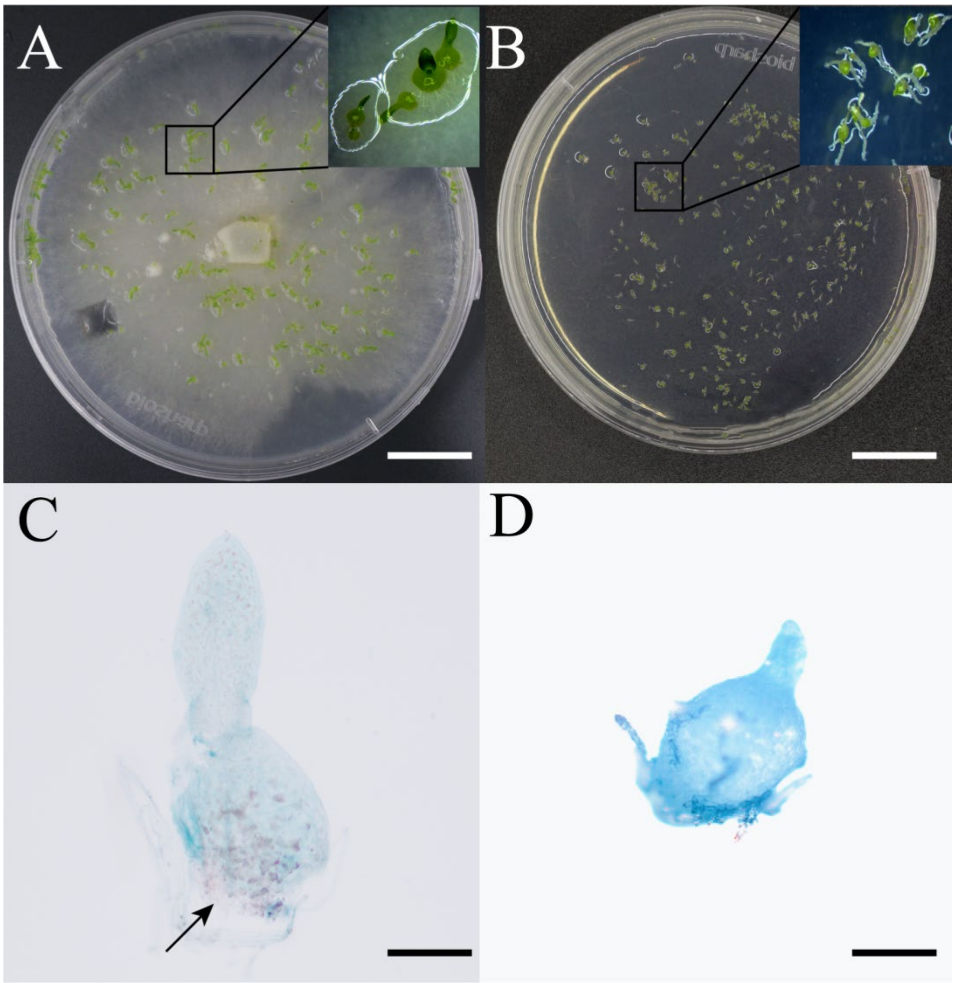

Fig. S1 Germination of *B. striata* protocorms and colonization by the mycorrhizal fungal strain bj1. A: *B. striata* protocorms grown symbiotically with bj1 (bar = 1 cm); B: *B. striata* protocorms grown asymbiotically (bar = 1 cm); C: Intracellular colonization by bj1 within a *B. striata* protocorm (arrow) (bar = 50 μm); D: Control *B. striata* protocorm without bj1 colonization (bar = 50 μm).

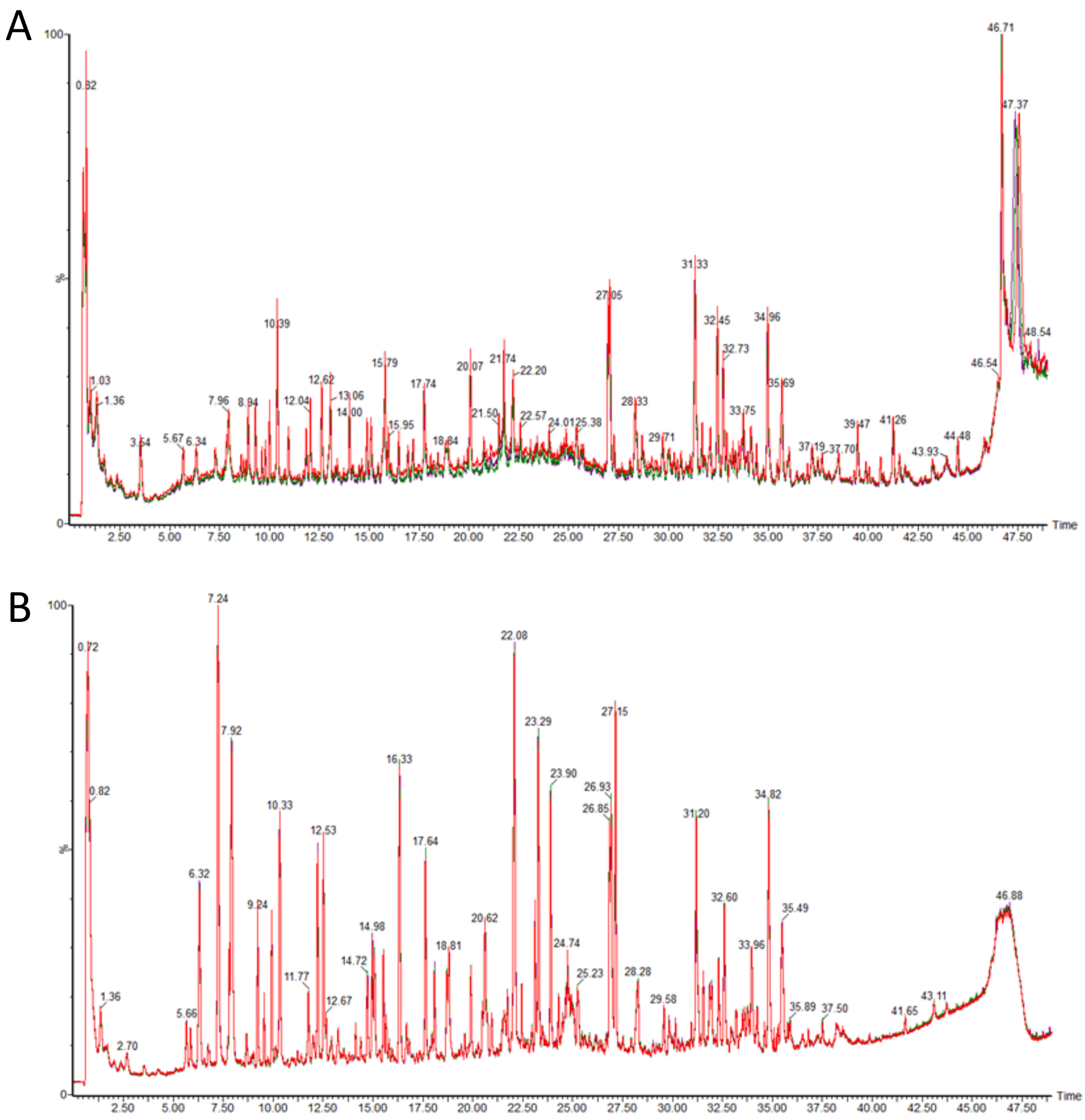

Figure S2 Total ions current in *B. striata*; A: the total ion current diagram of positive ion mode; B: the total ion current diagram of the negative ion mode.

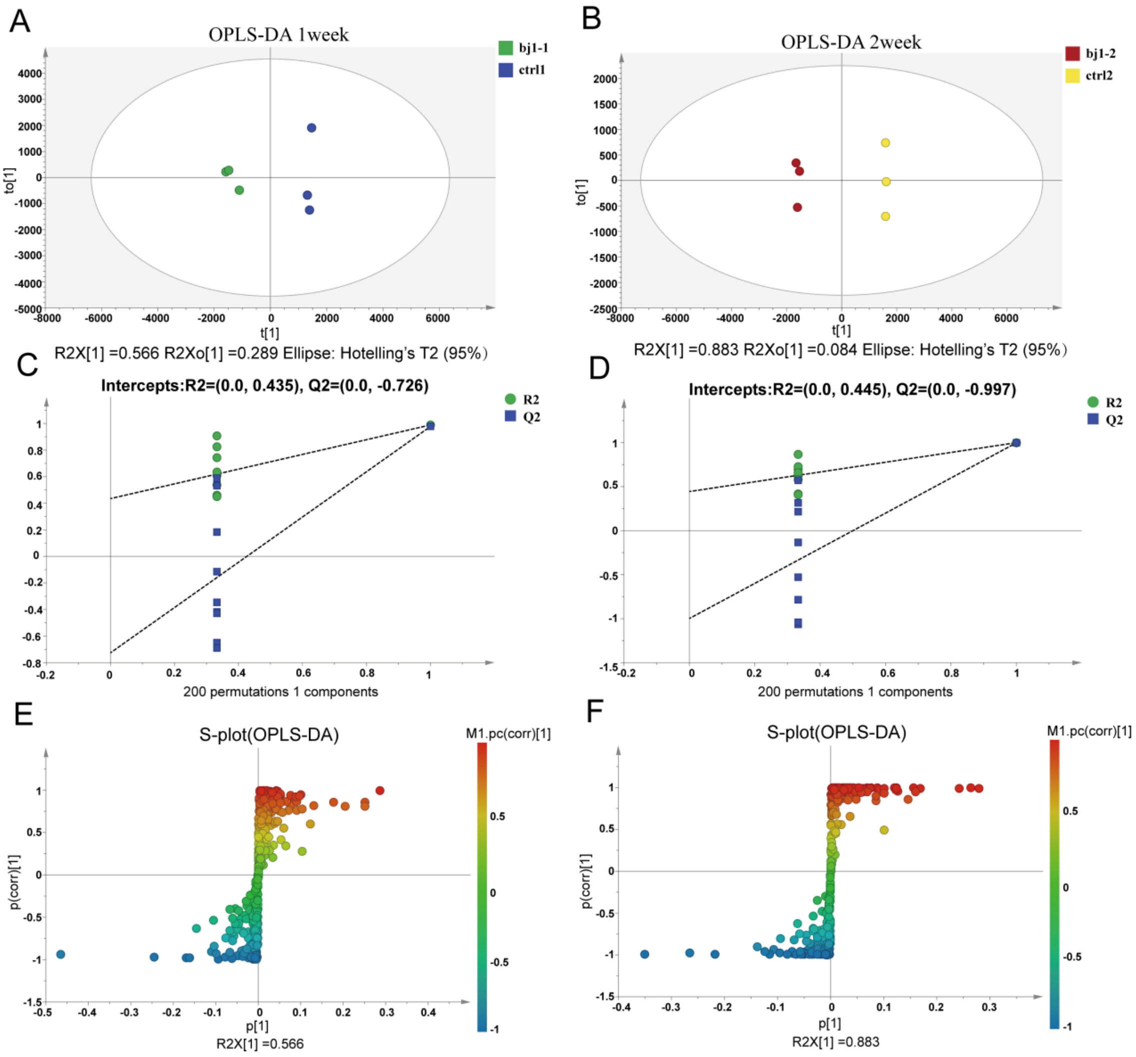

Fig. S3 OPLS-DA, displacement test and S-Plot of samples from the bj1 group and control group at 1 week and 2 week. A: The OPLS-DA score chart of the 1 week; B: The OPLS-DA score chart of the 2 week; C: The displacement test chart of the 1 week; D: The displacement test chart of the 2 week; E: The S-Plot of the 1 week; F: The S-Plot of the 2 week

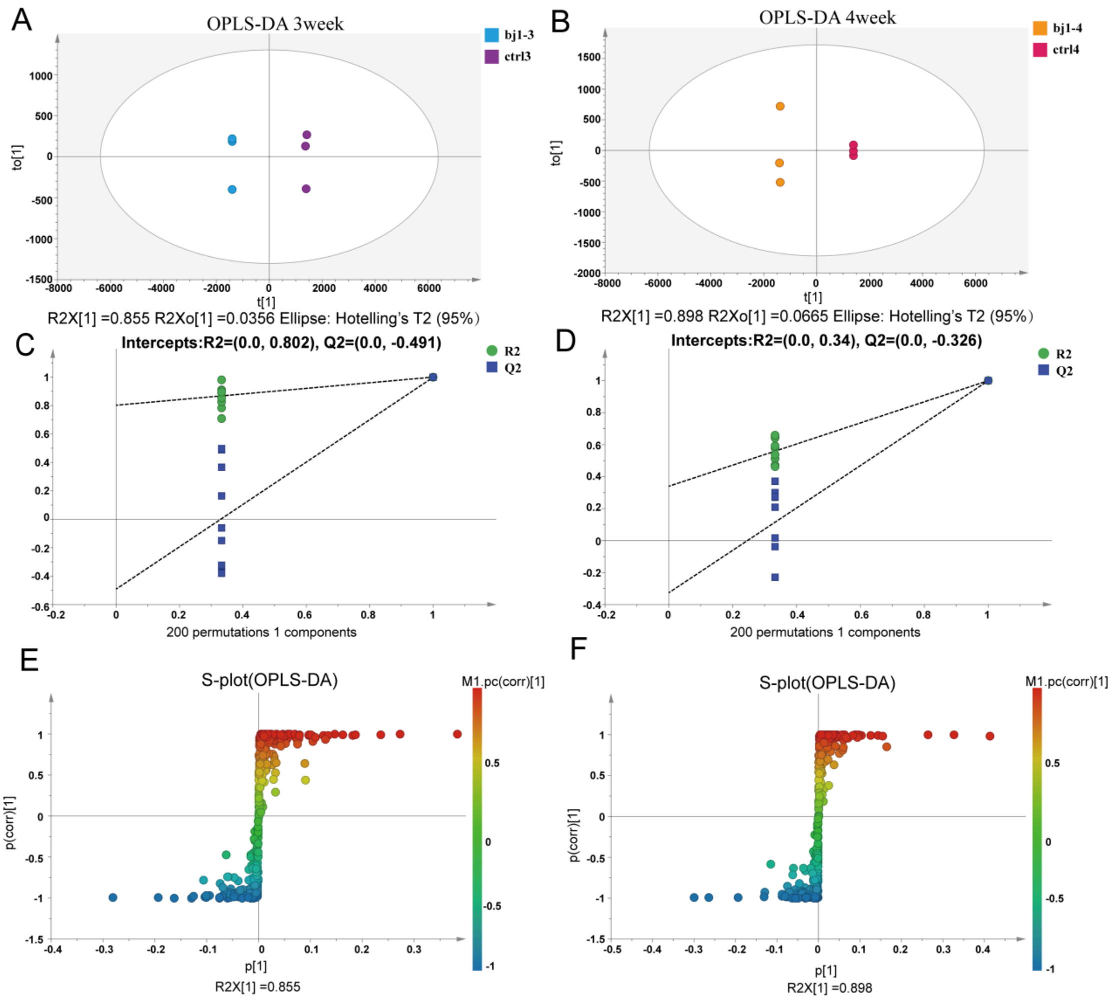

Fig. S4 OPLS-DA, displacement test and S-Plot of samples from the bj1 group and control group at 3 week and 4 week; A: The OPLS-DA score chart of the 3 week； B: The OPLS-DA score chart of the 4 week; C: The displacement test chart of the 3 week； D: The displacement test chart of the 4 week； E: The S-Plot of the 3 week； F: The S-Plot of the 4week

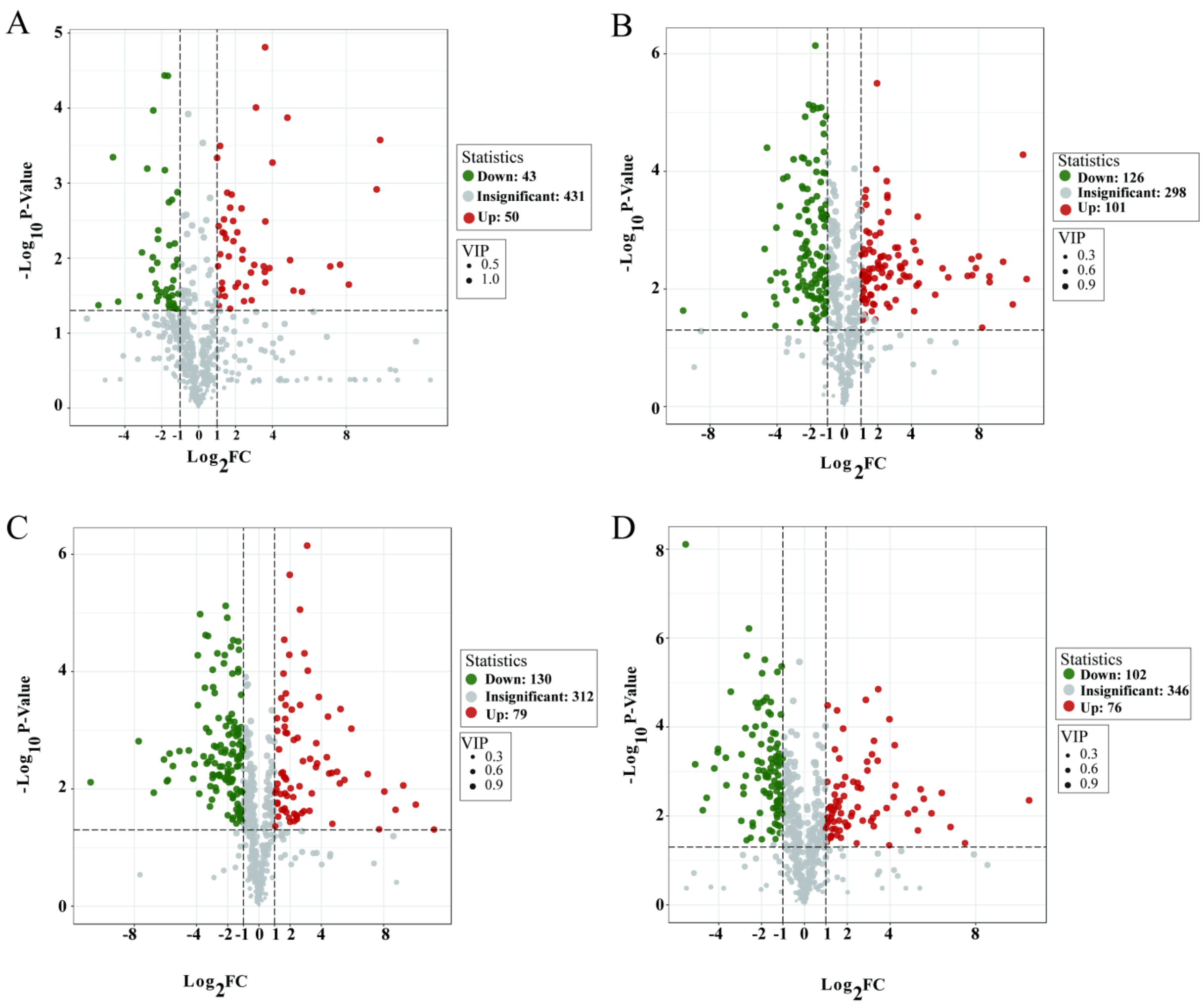

Fig. S5 Volcanic map analysis of different metabolites between bj1 group and control group; A-D were the differential metabolite volcano maps of week 1, week 2, week 3 and week 4, respectively. Each point in the volcano map represents a metabolite, the abscissa represents the quantitative difference in the two samples multiplied by the logarithm of the metabolite, and the ordinate represents the level of significance of the differential metabolite. The green/red dots in the figure indicate down-regulated/up-regulated differential metabolites, respectively.

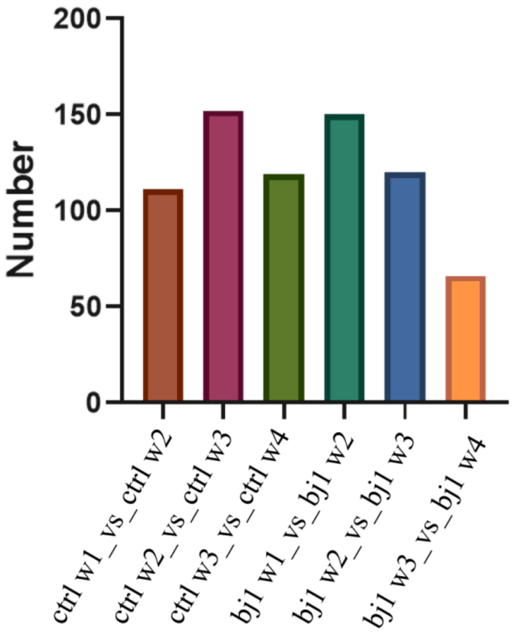

Fig. S6 Changes in DAMs at different time points in the bj1 and control groups.

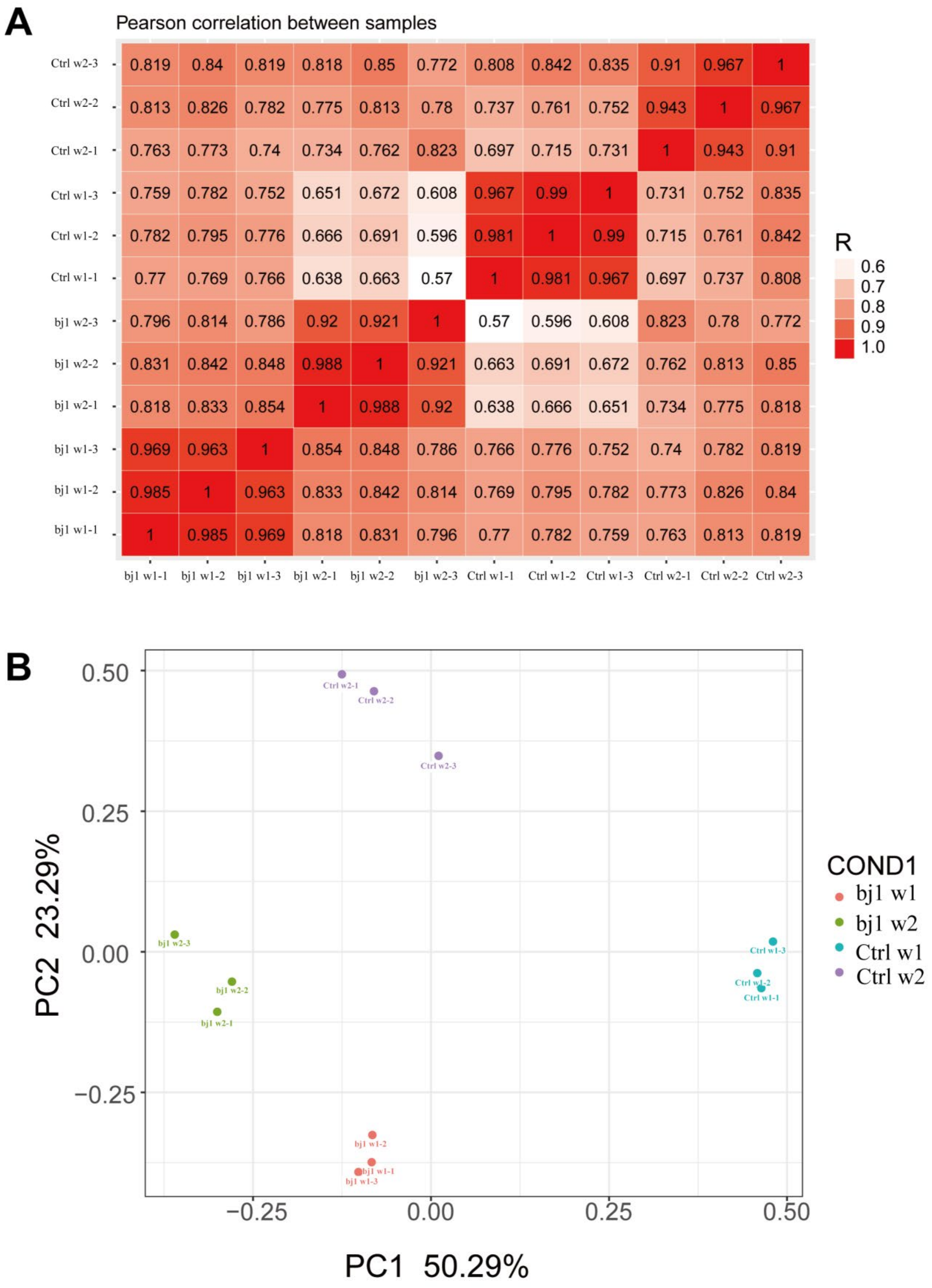

Fig. S7 Pearson correlation coefficient and PCA analysis diagram; A: Pearson correlation coefficient. The horizontal and vertical coordinates are for each sample respectively, and the color depth indicates the correlation coefficient size of the two samples. The closer it is to red (the closer the coefficient is to 1) the greater the correlation; The closer it is to white, the less correlated it is; B: PCA principal component analysis diagram.

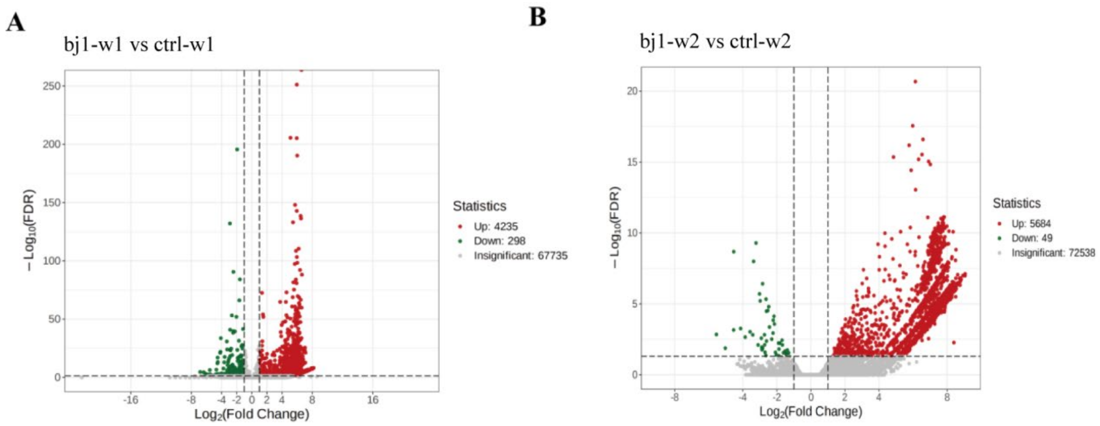

Fig. S8 Transcriptome analysis of *B. striata* seeds after exclusion of genes from the transcriptome of bj1 strain. A: Volcano map of DEGs in the (bj1 w1 vs ctrl w1) group; B: Volcano map of DEGs in the (bj1 w2 vs ctrl w2) group;

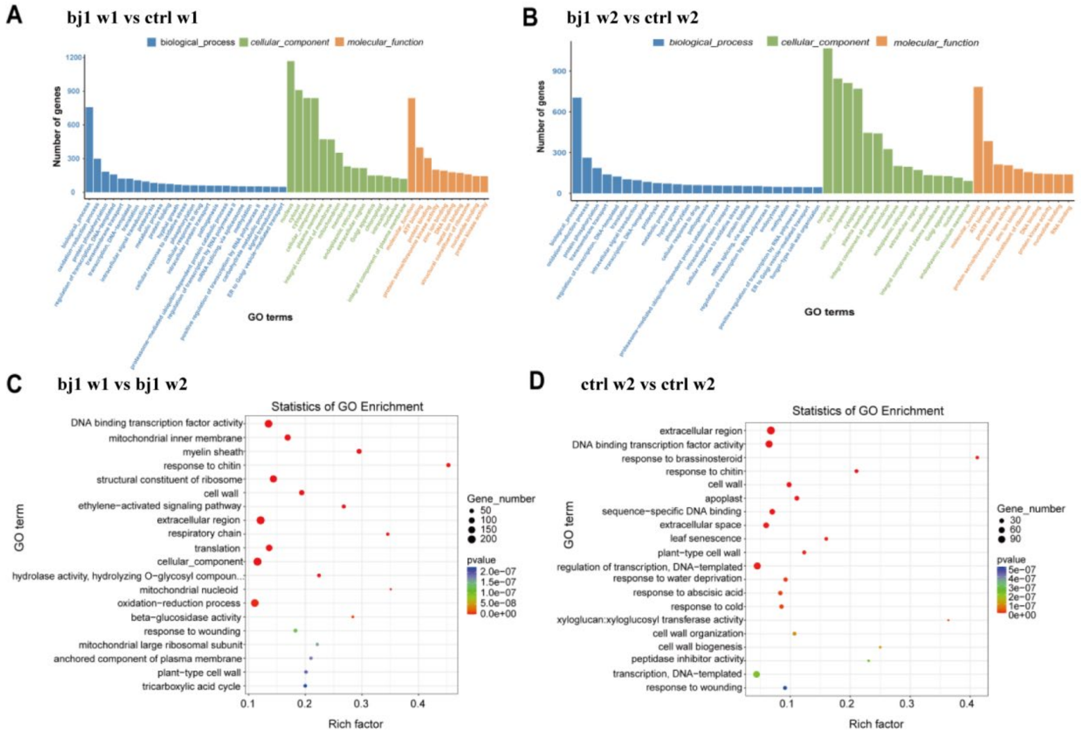

Fig. S9 GO enrichment analysis of differential genes between the bj1 group and the control group. A: (bj1 w1 vs ctrl w1) group GO enrichment; B: (bj1 w2 vs ctrl w2) group GO enrichment; C: (bj1 w1 vs bj1 w2) group GO enrichment; D: (ctrl w1 vs ctrl w2) group GO enrichment;

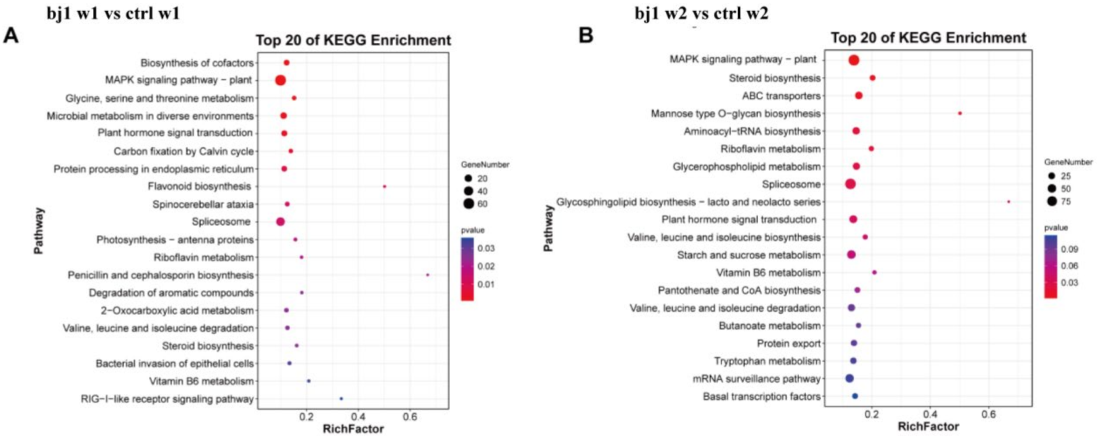

Fig. S10 KEGG analysis of *B. striata* seeds after exclusion of genes from the transcriptome of bj1 strain.

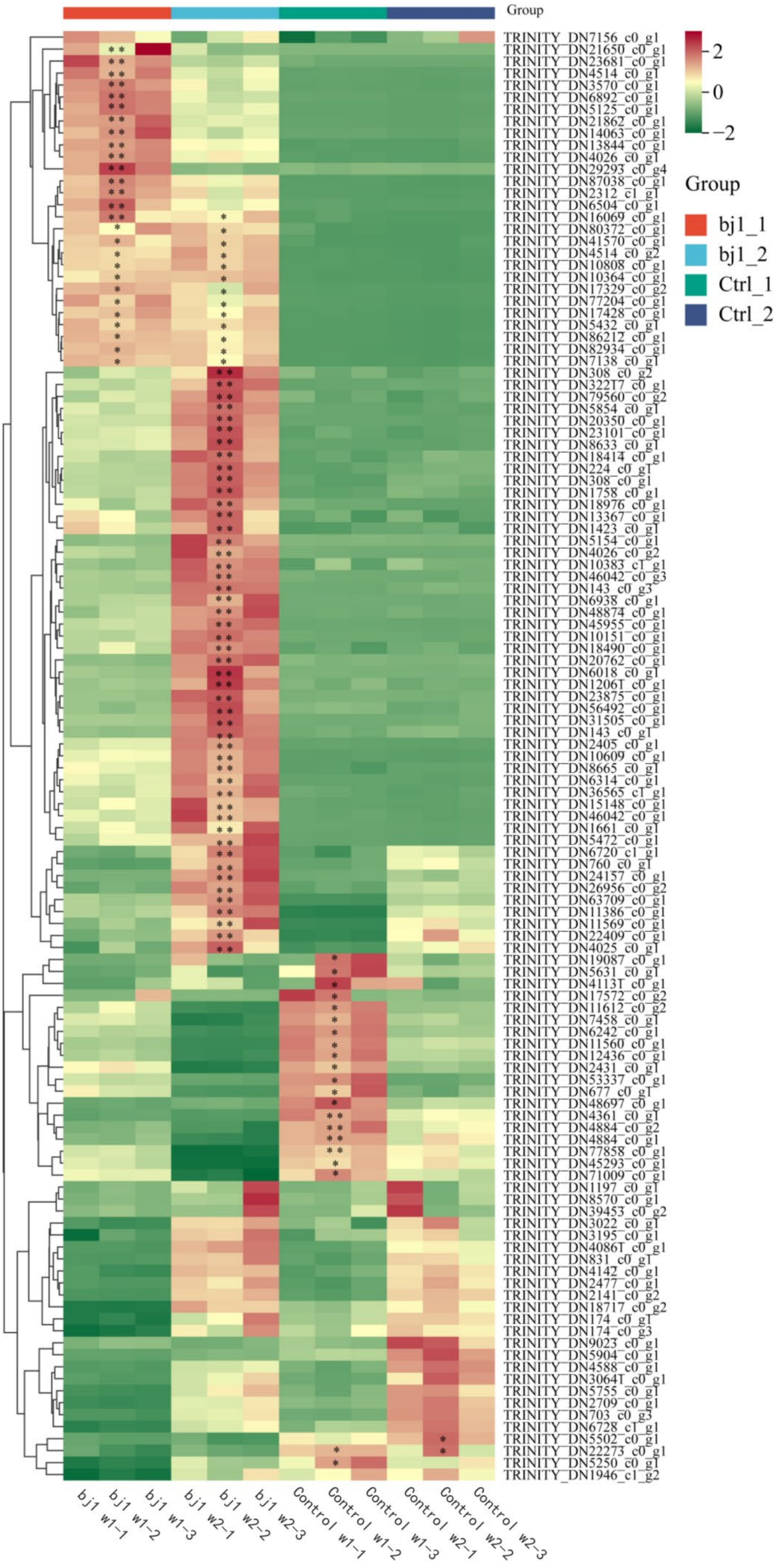

Group
2
0
−2
Group
bj1_1
bj1_2
Ctrl_1
Ctrl_2
TRINITY_DN7156_c0_g1
TRINITY_DN21650_c0_g1
TRINITY_DN23681_c0_g1
TRINITY_DN4514_c0_g1
TRINITY_DN3570_c0_g1
TRINITY_DN6892_c0_g1
TRINITY_DN5125_c0_g1
TRINITY_DN21862_c0_g1
TRINITY_DN14063_c0_g1
TRINITY_DN13844_c0_g1
TRINITY_DN4026_c0_g1
TRINITY_DN29293_c0_g4
TRINITY_DN87038_c0_g1
TRINITY_DN2312_c1_g1
TRINITY_DN6504_c0_g1
TRINITY_DN16069_c0_g1
TRINITY_DN80372_c0_g1
TRINITY_DN41570_c0_g1
TRINITY_DN4514_c0_g2
TRINITY_DN10808_c0_g1
TRINITY_DN10364_c0_g1
TRINITY_DN17329_c0_g2
TRINITY_DN77204_c0_g1
TRINITY_DN17428_c0_g1
TRINITY_DN5432_c0_g1
TRINITY_DN86212_c0_g1
TRINITY_DN82934_c0_g1
TRINITY_DN7138_c0_g1
TRINITY_DN308_c0_g2
TRINITY_DN32217_c0_g1
TRINITY_DN79560_c0_g2
TRINITY_DN5854_c0_g1
TRINITY_DN20350_c0_g1
TRINITY_DN23101_c0_g1
TRINITY_DN8633_c0_g1
TRINITY_DN18414_c0_g1
TRINITY_DN224_c0_g1
TRINITY_DN308_c0_g1
TRINITY_DN1758_c0_g1
TRINITY_DN18976_c0_g1
TRINITY_DN13367_c0_g1
TRINITY_DN1423_c0_g1
TRINITY_DN5154_c0_g1
TRINITY_DN4026_c0_g2
TRINITY_DN10383_c1_g1
TRINITY_DN46042_c0_g3
TRINITY_DN143_c0_g3
TRINITY_DN6938_c0_g1
TRINITY_DN48874_c0_g1
TRINITY_DN45955_c0_g1
TRINITY_DN10151_c0_g1
TRINITY_DN18490_c0_g1
TRINITY_DN20762_c0_g1
TRINITY_DN6018_c0_g1
TRINITY_DN12061_c0_g1
TRINITY_DN23875_c0_g1
TRINITY_DN56492_c0_g1
TRINITY_DN31505_c0_g1
TRINITY_DN143_c0_g1
TRINITY_DN2405_c0_g1
TRINITY_DN10609_c0_g1
TRINITY_DN8665_c0_g1
TRINITY_DN6314_c0_g1
TRINITY_DN36565_c1_g1
TRINITY_DN15148_c0_g1
TRINITY_DN46042_c0_g1
TRINITY_DN1661_c0_g1
TRINITY_DN5472_c0_g1
TRINITY_DN6720_c1_g1
TRINITY_DN760_c0_g1
TRINITY_DN24157_c0_g1
TRINITY_DN26956_c0_g2
TRINITY_DN63709_c0_g1
TRINITY_DN11386_c0_g1
TRINITY_DN11569_c0_g1
TRINITY_DN22409_c0_g1
TRINITY_DN4025_c0_g1
TRINITY_DN19087_c0_g1
TRINITY_DN5631_c0_g1
TRINITY_DN41131_c0_g1
TRINITY_DN17572_c0_g2
TRINITY_DN11612_c0_g2
TRINITY_DN7458_c0_g1
TRINITY_DN6242_c0_g1
TRINITY_DN11560_c0_g1
TRINITY_DN12436_c0_g1
TRINITY_DN2431_c0_g1
TRINITY_DN53337_c0_g1
TRINITY_DN677_c0_g1
TRINITY_DN48697_c0_g1
TRINITY_DN4361_c0_g1
TRINITY_DN4884_c0_g2
TRINITY_DN4884_c0_g1
TRINITY_DN77858_c0_g1
TRINITY_DN45293_c0_g1
TRINITY_DN71009_c0_g1
TRINITY_DN1197_c0_g1
TRINITY_DN8570_c0_g1
TRINITY_DN39453_c0_g2
TRINITY_DN3022_c0_g1
TRINITY_DN3195_c0_g1
TRINITY_DN40861_c0_g1
TRINITY_DN831_c0_g1
TRINITY_DN4142_c0_g1
TRINITY_DN2477_c0_g1
TRINITY_DN2141_c0_g2
TRINITY_DN18717_c0_g2
TRINITY_DN174_c0_g1
TRINITY_DN174_c0_g3
TRINITY_DN9023_c0_g1
TRINITY_DN5904_c0_g1
TRINITY_DN4588_c0_g1
TRINITY_DN30641_c0_g1
TRINITY_DN5755_c0_g1
TRINITY_DN2709_c0_g1
TRINITY_DN703_c0_g3
TRINITY_DN6728_c1_g1
TRINITY_DN5502_c0_g1
TRINITY_DN22273_c0_g1
TRINITY_DN5250_c0_g1
TRINITY_DN1946_c1_g2
bj1 w1-1
bj1 w1-2
bj1 w1-3
bj1 w2-1
bj1 w2-2
bj1 w2-3
Control w1-1
Control w1-2
Control w1-3
Control w2-1
Control w2-2
Control w2-3

Fig. S11 heat map analysis of all Starch and sucrose metabolism detected and associated DEGs; each color block in the heat map represents the relative expression of the gene at the corresponding location, and the green/red block in the figure represents the down-regulated/ up-regulated differential genes respectively. The darker the color, the higher the content, and vice versa. Most of the DEGs exhibited significantly higher expression levels in the symbiotic group compared to the control group. The independent sample t-test was employed to assess the statistical significance of differences between the two groups within the same week, *: $p < 0.05$, **: $p < 0.01$.

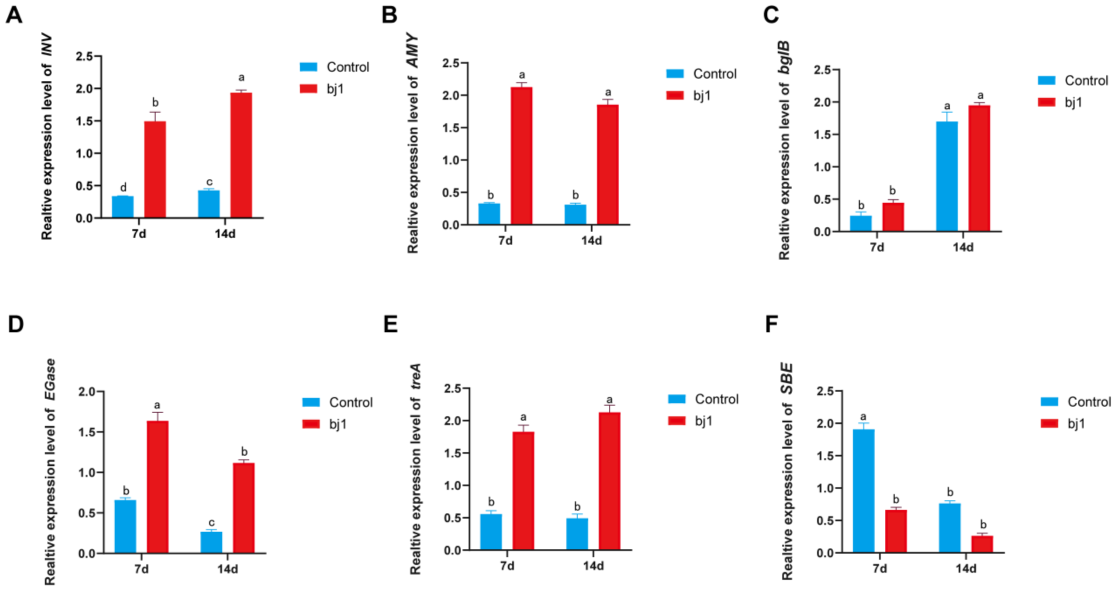

Fig. S12 qPCR verification of the expression levels of starch and sucrose metabolic pathways genes in *B. striata* protocorms. Significant differences between groups are indicated by different letters (a, b, c and d), $p<0.05$, by two-way ANOVA with Tukey's post-hoc test.
