# Supplementary material for: Deciphering Molecular Pathways of Bletilla striata Seeds Symbiotic Germination with Tulasnella sp. bj1
Source: Microorganisms. 2026 Jan 13;14(1):174. doi: 10.3390/microorganisms14010174 (PMC12844467; doi:10.3390/microorganisms14010174)
Supplement: Supplementary file 1 [file microorganisms-14-00174-s001.zip › Supplementary Table 1-2026.1.1.pdf]

Table S1 : *B. striata* genes and primers

	Gene	Forward primer	Reverse primer
Genes related to flavonoid biosynthesis	(GAPDH)TRINITY_DN6419_c0_g1	ATCAAGCAAGGACTGGAGGG	GGCAGACAAATGTATGGTGGC
	(PAL1)TRINITY_DN33508_c1_g2	GAAGGAGGTTGGTGCAGGTT	ACACAGATTGGCAAAGGAGC
	（C4H）TRINITY_DN64944_c1_g1	TCAGCGGGAAGAAGCTCAAG	CTTCTTGGCAAGGGTGGCTA
	（4CL）TRINITY_DN1091_c0_g1	CCTCCGAAACCCTCACCTTC	AGAAAGAGACGGCGAAGTGG
	（CHS）TRINITY_DN3408_c0_g1	GCCTTGATTGGGGTGTGTTG	CAAGCCCTATAGGTTGGAGTACA
	（CHI）TRINITY_DN13800_c1_g1	TCGGAGTGTATTTGGAGGCG	CTCGTCAGCTTCTCGAAGGG
	（FLS）TRINITY_DN28456_c0_g1	GAGGTTCAGGGGAAGAAGGC	ACTTTCCTGTAATCGGGCGG
	（F3H）TRINITY_DN4032_c0_g1	TTCAAGAACGCGGACCATCA	GCTCCAGGTCTCTGCTCATC
IAA biosynthesis	(TAA1)TRINITY_DN270_c0_g1	GGCCAATGGGAAGCATTTGG	TCATGCACTTTTCAACGGCG
	(YUCCA)TRINITY_DN1461_c0_g1	TCATGGTAGCCTCTACCGCT	GAAAAGCCCTAGGATCGCCA
	(TDC)TRINITY_DN4378_c0_g1	CACTGGCAGAGCCCAAACTA	GTTGCAGCAGGGGAGGTTAT
	(NtAMI1)TRINITY_DN1040_c0_g1	TGATGAACTCAGTGGTGGGC	TAAAGCTCTCTGCCGCCTTC
	(NIT2)TRINITY_DN7670_c0_g1	ACAGTATCAAAGGCGTGGGG	AGAAGCACAACAGAGCCGAA
JA biosynthesis	(LOX)TRINITY_DN753_c1_g1	CTAGGCGTTGTCGAACTGGT	CTCGGCGATCATCTCTTGCA
	(AOC)TRINITY_DN5174_c0_g1	CCTTTTCCCCTTCCCCCATC	AGAGATTGGAAGGGTTGGCG
	(OPR)TRINITY_DN4182_c0_g1	TAGAGAATCGCTGCCGCTTT	TCAGAGTAGGCGGCAAATGG
starch and sucrose metabolic	(INV)TRINITY_DN18414_c0_g1	ACAGGATGACTGGGCAAAGG	AGCTAACCACCACATCAGCC
	（bglB）TRINITY_DN53337_c0_g1	GCCAGGAGCCAACTAACAGT	CCCTCGACCATCTTCGTTGT
	（SBE）TRINITY_DN71009_c0_g1	GGCAGCATGGATGGCAAAAA	GGTATGCCAATGTCCAGCCT
	(AMY)TRINITY_DN10364_c0_g1	GAACAATGCTGGGTCGGAGA	ACGAACGTGCTTGACGGTAT
	（treA）TRINITY_DN5125_c0_g1	TGATCCCACTGAACCACACG	GTAGATCCTGCCTCCGTTCG
	(EGase)TRINITY_DN4588_c0_g1	ATATGGACTACCCACGGCCT	CTAGCTCTCTGCATCCTCGC

Table S2 Cumulative interpretation rate of OPLS-DA model between samples

Comparison group	R^2X	R^2Y	Q^2
1week bj1-1 vs control 1	0.855	0.989	0.977
2week bj1-2 vs control 2	0.967	0.999	0.997
3week bj1-3 vs control 3	0.891	1	0.998
4week bj1-4 vs control 4	0.964	1	1

Table S4 Statistical table of raw transcriptome data of *B. striata* protocorms at 1-2 weeks

samples	raw_base（G）	raw_sequences	Valid_bases (G)	Valid_sequences	q20（%）	q30 (%)	GC (%)	Valid %
bj1 1-1	5.34	35573704	4.81	32589896	98.63	95.61	48.44	91.61
bj1 1-2	5.43	36208946	4.89	33181792	98.61	95.56	48.57	91.64
bj1 1-3	6.69	44601388	6.04	40977696	98.89	96.41	48.72	91.88
bj1 2-1	5.95	39683412	5.41	36724350	98.89	96.41	48.79	92.54
bj1 2-2	6.84	45630582	6.20	42094784	98.86	96.33	48.43	92.25
bj1 2-3	5.51	36736378	5.11	34792764	98.48	95.19	47.94	94.71
control 1-1	5.82	38772404	5.23	35508192	98.87	96.34	47.14	91.58
control 1-2	6.71	44744140	6.03	40906604	98.84	96.26	47.49	91.42
control 1-3	6.26	41754204	5.64	38249958	98.88	96.37	47.34	91.61
control 2-1	5.94	39575170	5.48	37351988	98.44	95.10	46.35	94.38
control 2-2	6.53	43521994	6.02	40987970	98.54	95.39	46.35	94.18
control 2-3	6.11	40751314	5.35	36571406	98.89	96.42	47.16	89.74
